# Mannose-Functionalized Chitosan-TPGS/Tween 80 Nanocarriers for Macrophage Targeting: Enhanced Piperine Delivery to Potentiate Anti-Inflammatory and Antioxidant Therapy

**DOI:** 10.3390/antiox15050559

**Published:** 2026-04-28

**Authors:** Abdullah Mohammed Ayedh Al Adhreai, Johnson Retnaraj Samuel Selvan Christyraj, Prathiba Gnanasekaran, Hemanth P. K. Sudhani, Haorongbam Joldy Devi, Yumnam Asha Devi, Maharshi Bhaswant

**Affiliations:** 1 Regeneration and Stem Cell Biology Lab, Centre for Molecular and Nanomedical Sciences, International Research Centre, Sathyabama Institute of Science and Technology, Chennai 600119, Tamil Nadu, India; raedmbc2016@gmail.com; 2 Department of Microbiology, Sathyabama Dental College and Hospital, Chennai 600119, Tamil Nadu, India; brightstart2011@yahoo.com (P.G.); joldyhaorongbam@gmail.com (H.J.D.); yumnamashalang@gmail.com (Y.A.D.); 3 School of Biosciences and Technology, SRM Institute of Science and Technology, Tiruchirappalli Campus, Tiruchirappalli 621105, Tamil Nadu, India; sudhanip@srmist.edu.in

**Keywords:** piperine nanodelivery, mannosylated chitosan nanoparticles, TPGS/Tween 80 stabilization, macrophage-targeted delivery, anti-inflammatory activity, antioxidant properties

## Abstract

Piperine (PIP), a plant alkaloid with anti-inflammatory and antioxidant effects, has poor solubility and bioavailability, limiting its therapeutic potential in macrophage-mediated inflammatory and oxidative stress conditions. Despite various nanocarrier systems being explored for bioactive compounds, the specific combination of mannose-functionalized chitosan with dual stabilizers (TPGS and Tween 80) for enhanced macrophage targeting and piperine delivery has not been investigated. We hypothesized that this novel formulation would significantly enhance piperine solubility, macrophage uptake, and anti-inflammatory/antioxidant effects compared to conventional systems, while modulating apoptosis-related pathways. This study evaluated targeted and non-targeted nanoparticles synthesized by ionic gelation and emulsification using RAW 264.7 and THP-1 macrophages. FTIR, UV–Vis, XRD, and CHNS confirmed mannose conjugation, while SEM, TEM, and AFM revealed morphology. Physicochemical properties were assessed by DLS, encapsulation efficiency (EE%), drug loading (DL%), and stability. Biological evaluations included drug release, cytotoxicity (MTT), apoptosis analysis (Annexin V–FITC/PI staining), cellular uptake (fluorescence microscopy with coumarin-6), anti-inflammatory assays (extracellular and intracellular NO inhibition, cytokine suppression), antioxidant activity (DPPH, ABTS, FRAP, TAC), intracellular ROS/RNS, and apoptosis-related markers. Targeted nanoparticles showed larger mean size (162 nm) versus non-targeted ones (78 nm). EE% was 82% (targeted) and 92% (non-targeted). Both demonstrated sustained 72 h release. Cellular uptake was significantly greater for targeted nanoparticles. Both formulations reduced NO and pro-inflammatory cytokines, regulated apoptosis-associated markers, and induced controlled apoptosis at higher concentrations, with stronger effects observed for targeted particles. Antioxidant activity increased dose-dependently, with targeted nanoparticles showing superior intracellular ROS/RNS suppression. This novel multi-functional platform efficiently encapsulates PIP, enhances macrophage targeting, modulates apoptosis pathways, and demonstrates superior therapeutic promise for inflammation-related disorders.

## 1. Introduction

Chronic inflammation and oxidative stress are fundamental drivers of many degenerative and infectious diseases, including rheumatoid arthritis, osteoarthritis (OA), inflammatory bowel disease, and neurodegenerative disorders. These conditions are characterized by excessive production of pro-inflammatory cytokines and reactive oxygen/nitrogen species (ROS/RNS), leading to tissue injury, cellular dysfunction, and disease progression. The interplay between persistent inflammation, oxidative stress, and dysregulated signaling pathways such as NF-κB and MAPK makes these disorders complex and multifactorial [1,2,3,4,5]. Current therapeutic strategies primarily focus on symptomatic management, highlighting the urgent need for interventions that target both inflammatory and redox imbalances at the cellular level [6,7,8].

Macrophages play a pivotal role in driving inflammation and oxidative stress across multiple disease contexts [9]. Activated macrophages release high levels of tumor necrosis factor-alpha (TNF-α), interleukin-6 (IL-6), and interleukin-1 beta (IL-1β), along with ROS and RNS, which amplify oxidative damage and sustain chronic inflammation. These mediators not only exacerbate local tissue injury but also create a feed-forward cycle of cytokine release, ROS/RNS generation, and cellular apoptosis. Therefore, macrophage-targeted therapies that can suppress pro-inflammatory signaling while enhancing antioxidant defenses represent a promising strategy for managing a wide range of inflammation-associated disorders, with OA serving as one clinically relevant example [10,11,12,13,14,15,16].

Piperine (PIP), a naturally occurring alkaloid derived from black pepper (Piper nigrum), has emerged as a promising therapeutic candidate due to its potent anti-inflammatory and antioxidant properties. Studies have demonstrated that piperine can effectively modulate inflammatory signaling pathways, reduce ROS/RNS production, and protect against oxidative damage in diverse disease models [17,18,19,20,21,22]. However, its clinical translation is limited by poor water solubility, low bioavailability, and inadequate intracellular delivery to target cells such as macrophages [23,24,25]. To address these limitations, nanoparticle-based drug delivery systems have been extensively explored, offering advantages such as enhanced solubility, improved stability, controlled release, and targeted uptake. Nanoparticles can increase intracellular concentrations of hydrophobic drugs like piperine, protect them from metabolic degradation, and sustain therapeutic activity in macrophages and other target cells [26,27,28,29,30,31].

Chitosan, a biocompatible and biodegradable polysaccharide, has been widely employed as a nanoparticle matrix due to its positive surface charge, mucoadhesive properties, and intrinsic biocompatibility. These features promote strong interactions with cell membranes and facilitate endocytic internalization [32,33]. Moreover, chitosan nanoparticles themselves have demonstrated intrinsic anti-inflammatory and antioxidant effects, further enhancing the therapeutic potential of encapsulated drugs [34,35]. Surface modification of chitosan nanoparticles with mannose enables active targeting of macrophages via mannose receptor-mediated endocytosis, leading to increased intracellular accumulation of therapeutic agents [36,37]. Previous studies confirm that mannose-decorated nanoparticles improve selective uptake in macrophages and other high-mannose-expressing cells, thereby enhancing drug efficacy while minimizing systemic toxicity [38].

Additionally, surfactants and solubilizing agents such as Tween 80 and d-α-tocopheryl polyethylene glycol succinate (TPGS) are commonly incorporated into nanoparticle formulations [33,39]. Tween 80 enhances nanoparticle dispersion and stability, reducing aggregation and improving cellular uptake [40,41,42,43], whereas TPGS, an FDA-approved amphiphilic excipient, improves solubility, prevents aggregation, enhances uptake, and inhibits efflux transporters that limit intracellular drug retention. TPGS-containing nanoparticles have been shown to increase the bioavailability of hydrophobic drugs like piperine and further potentiate their anti-inflammatory and antioxidant activities in target cells [33,44,45,46,47,48].

Previous research has explored chitosan nanoparticles for macrophage targeting [49], mannose-functionalized carriers for improved receptor-mediated uptake [50], and TPGS/Tween 80 stabilization for enhancing drug solubility [33,40]. However, despite the individual success of these approaches, no study has investigated their synergistic combination for macrophage-targeted piperine delivery. This represents a significant knowledge gap, as the integration of mannose targeting, chitosan biocompatibility, and dual TPGS/Tween 80 stabilization could potentially address multiple delivery challenges simultaneously while maximizing therapeutic efficacy. We hypothesize that this novel multi-functional approach of mannosylated chitosan nanoparticles stabilized with TPGS and Tween 80 can synergistically improve piperine solubility, enhance selective uptake by macrophages (RAW 264.7 and THP-1), and significantly potentiate its anti-inflammatory and antioxidant effects beyond individual component systems. Accordingly, this study aims to develop and characterize these novel nanoparticles, evaluate their physicochemical and morphological properties, and assess their in vitro macrophage uptake, anti-inflammatory, and antioxidant activities. Successful demonstration of this targeted nanoparticle platform could provide a promising strategy for macrophage-directed therapy in OA and other inflammation-driven conditions.

## 2. Materials and Methods

### 2.1. Chemicals and Reagents

Chitosan (low molecular weight; Cat. No. 9012-76-4), coumarin-6 (CM6; Cat. No. C8660), sodium tripolyphosphate (TPP; Cat. No. 7758-29-4), D-mannose (Cat. No. M6020), 2′,7′dichlorodihydrofluorescein diacetate (DCFH-DA; Cat. No. D6883), lipopolysaccharide (LPS; Cat. No. L2630), interferon-gamma (IFN-γ; Cat. No. I17001), and 4-amino-5-methylamino-2′,7′-difluorofluorescein diacetate (DAF-FM DA; Cat. No. D23844) were purchased from Sigma-Aldrich (Bangalore, India). RPMI-1640 medium (Cat. No. R8758), DMEM medium (Cat. No. D6429), penicillin–streptomycin solution (Cat. No. P4333), L-glutamine (Cat. No. G7513), acetone (Cat. No. 67-64-1), chloroform (Cat. No. 67-66-3), and 1 kDa cellulose dialysis tubing was obtained from HiMedia Laboratories (Mumbai, India). D-α-tocopheryl polyethylene glycol 1000 succinate (TPGS), Tween-80, and phorbol 12-myristate 13-acetate (PMA) were kindly provided by the donor. All chemicals and reagents used in the study were of analytical grade and used without further purification. Piperine was extracted and purified from *Piper nigrum* fruits (Kanyakumari, Tamil Nadu, India) using standard phytochemical methods, as described in our previously published work [51].

### 2.2. Cell Lines

Murine macrophages (RAW 264.7) and human monocytes (THP-1) were obtained from the National Centre for Cell Science (NCCS, Pune, India). Cells were maintained according to the supplier guidelines under standard culture conditions.

### 2.3. Synthesis of Non-Targeted and Mannose-Targeted Nanoparticles

Piperine-loaded chitosan-TPGS-Tween 80 nanoparticles (PIP-CTT-NPs) and mannose-conjugated piperine-loaded chitosan-TPGS-Tween 80 nanoparticles (PIP-MCTT-NPs) were prepared through a modified emulsification–ionic gelation process [52]. Initially, low-molecular-weight chitosan was dispersed in acetic acid (0.2% *v*/*v*) and adjusted to pH 5.2. The polymer was allowed to hydrate and dissolve under gentle stirring for approximately 5 h. Once a clear solution was obtained, Tween 80 (1% *v*/*v*) and TPGS were added, and the mixture was stirred continuously at 250 rpm for 24 h at room temperature. Separately, piperine was dissolved in ethanol and added dropwise to the chitosan dispersion under constant stirring. After 60 min, the mixture was probe-sonicated to promote the formation of micelle-like (mixed micellar) assemblies encapsulating piperine, followed by stirring for 15 h to ensure complete evaporation of ethanol. STPP dissolved in water was then added gradually to induce ionic cross-linking. After 30 min, large aggregates were removed by low-speed centrifugation, and nanoparticles were collected at high speed. The pellets were washed and gently resuspended.

**Figure 1:**
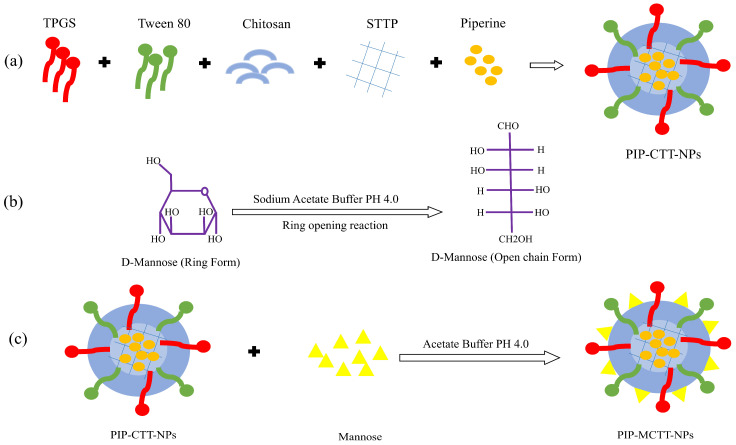
Schematic illustration of (**a**) preparation of piperine-loaded chitosan–TPGS–Tween 80 nanoparticles (PIP-CTT-NPs), (**b**) mannose conjugation reaction, and (**c**) mannose-conjugated piperine-loaded chitosan–TPGS–Tween 80 nanoparticles (PIP-MCTT-NPs).

For mannose-modified nanoparticles, D-mannose in acetate buffer (pH 4.0) was heated at 60 °C for 1 h to activate the aldehyde groups [53]. This solution was added to the PIP-CTT-NP suspension and stirred continuously for 72 h at room temperature to promote Schiff base formation (–N=CH–) between mannose aldehydes and the primary amines of chitosan. Unreacted mannose and small molecules were removed by dialysis using cellulose tubing with a 1 kDa molecular weight cut-off, and the purified nanoparticles were finally lyophilized. Coumarin-6-loaded chitosan-TPGS-Tween 80 nanoparticles (CM6-CTT-NPs) and mannose-conjugated coumarin-6-loaded chitosan-TPGS-Tween 80 nanoparticles (CM6-MCTT-NPs) were prepared using the same procedure, replacing piperine with coumarin-6 (Table 1; Figure 1 and Figure S1) [33].

### 2.4. Characterization of Non-Targeted and Mannose-Targeted Nanoparticles

#### 2.4.1. Chemical Structure and Conjugation Analysis (FTIR, UV–Vis, XRD, CHNS)

Fourier-transform infrared (FTIR) spectroscopy (Bruker, MA, USA) was employed to identify characteristic functional groups and to confirm piperine encapsulation as well as chitosan–mannose conjugation in the nanoparticle formulations. Samples of piperine (PIP), chitosan (CTS), mannose (MNS), PIP-CTT-NPs, and PIP-MCTT-NPs were finely ground with spectroscopic-grade potassium bromide (KBr) and compressed into pellets. FTIR spectra were recorded in the range of 4000–600 cm^−1^ using an FTIR spectrometer (Bruker, MA, USA) with a resolution of 4 cm^−1^ and 32 scans per sample [33,54].

Ultraviolet–visible (UV–Vis) spectroscopy was used to examine the electronic absorption behavior of free piperine and nanoparticle formulations and to further confirm successful drug encapsulation. Spectra were recorded between 200 and 600 nm using a UV–vis spectrophotometer (Lambda 25, PerkinElmer, Waltham, MA, USA). Piperine was dissolved in methanol, CTS and MNS were dissolved in deionized water, and nanoparticles were analyzed as aqueous dispersions. All spectra were baseline-corrected using the corresponding solvents as blanks [29,55].

X-ray diffraction (XRD) analysis was performed to evaluate the crystalline or amorphous nature of individual components and nanoparticle formulations. Powdered samples of PIP, CTS, MNS, PIP-CTT-NPs, and PIP-MCTT-NPs were analyzed using an X-ray diffractometer (PW 1730, Philips, Eindhoven, The Netherlands) equipped with Cu Kα radiation (λ = 1.5406 Å), operated at 40 kV and 30 mA. Diffraction patterns were recorded over a 2θ range of 5–60° with a step size of 0.02°. The crystallinity index (CrI) was calculated using Equation (1) [52].

Elemental (CHNS) analysis was conducted to confirm changes in elemental composition following nanoparticle formation and mannose conjugation. Carbon, hydrogen, nitrogen, and sulfur contents were measured using a CHNS analyzer (PerkinElmer 2400 Series II, Waltham, MA, USA) (PerkinElmer 2400 Series II, USA). The nitrogen content was further used to calculate the degree of substitution (DS) of mannose on chitosan in PIP-MCTT-NPs using Equation (2) [56]. Since PIP-CTT-NPs do not contain mannose, DS was not calculated for this formulation.

(1) $$\mathrm{CrI}\;(\%)=\frac{I_{c}-I_{a}}{I_{c}}\times 100$$

(2) $$\mathrm{DS}\;(\%)=\frac{N_{\mathrm{CTS}}-N_{\mathrm{PIP-MCTT-NPs}}}{N_{\mathrm{CTS}}}\times \frac{M_{\mathrm{unit}}}{M_{\mathrm{MNS}}}\times 100$$

where *I_c_* is the maximum intensity of the principal crystalline peak, *I_a_* is the intensity of the amorphous background, *N*_CTS_ is the nitrogen content (%) of chitosan, *N*_PIP-MCTT-NPs_ is the nitrogen content (%) of mannose-modified nanoparticles, *M*_unit_ is the molecular weight of the chitosan repeating unit (161 g/mol), and *M*_MNS_ is the molecular weight of the mannose residue (180 g/mol).

#### 2.4.2. Physicochemical Characterization (DLS, EE%, DL%, Stability)

Dynamic light scattering (DLS) was employed to determine the hydrodynamic diameter, polydispersity index (PDI), and zeta potential of non-targeted and mannose-targeted nanoparticles. Nanoparticle suspensions were diluted appropriately with distilled water to avoid multiple scattering effects and filtered through 0.22 µm syringe filters prior to measurement. Analyses were performed using a Zetasizer Nano S90 (Malvern Instruments, Malvern, UK) at 25 °C with a fixed scattering angle of 90°. Each sample was measured in triplicate (*n* = 3 independent measurements), and the results were reported as mean ± standard deviation [37].

Encapsulation efficiency (EE%) and drug loading (DL%) of piperine (PIP) and coumarin-6 (CM6) were determined using an indirect method. Following nanoparticle formation, samples were centrifuged to separate free (unencapsulated) drug from the nanoparticle pellet. The concentration of free PIP in the supernatant was quantified by UV–vis spectrophotometry at 342 nm, while CM6 concentration was measured using fluorescence spectroscopy (excitation 462 nm, emission 502 nm) using appropriate calibration curves [33]. EE% and DL% were calculated using Equations (3) and (4), respectively.

(3) $$\mathrm{EE}\;(\%)=\frac{\mathrm{Amount}\;\mathrm{of}\;\mathrm{drug}\;\mathrm{encapsulated}}{\mathrm{Total}\;\mathrm{amount}\;\mathrm{of}\;\mathrm{drug}\;\mathrm{added}}\times 100$$

(4) $$\mathrm{DL}\;(\%)=\frac{\mathrm{Amount}\;\mathrm{of}\;\mathrm{drug}\;\mathrm{encapsulated}}{\mathrm{Total}\;\mathrm{weight}\;\mathrm{of}\;\mathrm{nanoparticles}}\times 100$$

Nanoparticle stability was evaluated by storing lyophilized samples at 4 °C and 25 °C for 30 days. At predetermined time points (1, 15, and 30 days), samples were reconstituted in distilled water and analyzed for changes in particle size, PDI, and zeta potential using DLS. All measurements were conducted in triplicate to assess physicochemical stability over time [37].

#### 2.4.3. Morphological Characterization (SEM, TEM, AFM)

Scanning electron microscopy (SEM) was performed to evaluate the surface morphology of PIP-CTT-NPs and PIP-MCTT-NPs. Lyophilized nanoparticles were diluted twice, placed on glass slides, dried under vacuum, and sputter-coated with a thin gold–palladium layer (~10–15 nm) to prevent charging. Imaging was carried out using a ZEISS Supra 40 SEM (Carl Zeiss, Oberkochen, Germany) (Carl Zeiss, Germany) at 20 kV [33,37]. Transmission electron microscopy (TEM) was employed to examine nanoparticle shape and size distribution. Tenfold-diluted nanoparticle suspensions were drop-cast onto carbon-coated copper grids and allowed to air-dry. Images were captured using a Philips CM-12 TEM (Philips, Andover, MA, USA) at 80 kV. The particle size distributions obtained from TEM and SEM images were analyzed using ImageJ software (version 1.54g) [33]. Atomic force microscopy (AFM) was used to analyze surface topology and roughness. Nanoparticle suspensions were dried on clean glass coverslips at 25 °C for 24 h and imaged in tapping mode using a Solver P-47 PRO AFM (NT-MDT, Moscow, Russia) with silicon cantilevers (F_0_ = 241 kHz, ~41 N/m). Images were analyzed using dedicated software to assess particle uniformity and surface features [57].

### 2.5. In Vitro Biological Studies

#### 2.5.1. PIP Release Study

Drug release studies of PIP from non-targeted and targeted nanoparticles were carried out using the dynamic dialysis method, as described previously [58]. Briefly, 4 mL of nanoparticle suspension (2500 μg/mL) was placed in a pre-treated dialysis bag (MWCO 12–14 kDa) and immersed in 100 mL of PBS (pH 7.4) containing 0.5% DMSO to maintain sink conditions. The sealed setup was maintained at 37 °C under gentle agitation (100 rpm) in a thermostated shaker. At predetermined time intervals, 6 mL aliquots of the release medium were withdrawn and immediately replaced with an equal volume of fresh, pre-warmed PBS–DMSO solution to maintain a constant volume. Samples were filtered through a 0.45 µm syringe filter to remove particulate matter, and the concentration of released PIP was quantified by UV–vis spectrophotometry at 342 nm against a calibration curve prepared in the same release medium, as described previously.

#### 2.5.2. Cell Culture Conditions

THP-1 human monocytic leukemia cells (suspension) and RAW 264.7 murine macrophage-like cells (adherent) were cultured at 37 °C in a humidified incubator with 5% CO_2_. THP-1 cells were maintained in RPMI-1640 medium, and RAW 264.7 cells in high-glucose DMEM. In both cases, the medium was supplemented with 10% heat-inactivated fetal bovine serum, 1% penicillin–streptomycin, and 2 mM L-glutamine. Routine passaging was performed to sustain cell viability and ensure reproducibility across experiments [59,60].

#### 2.5.3. PMA Concentration Optimization for THP-1 Viability and Differentiation

To determine the optimal non-toxic PMA concentration for THP-1 differentiation, cells (1 × 10^5^/well) were seeded in 96-well plates and treated with PMA (0–400 ng/mL; final DMSO ≤ 0.1%) in triplicates, alongside untreated, vehicle, and blank controls. After 24 h, cell viability was assessed by MTT assay (0.5 mg/mL, 3 h), formazan crystals were dissolved in DMSO, and absorbance was measured at 570 nm. Cell viability (%) was calculated as in Equation (5). The optimal PMA concentration was defined as the lowest dose inducing morphological adherence and flattening while maintaining ≥80–90% viability [61].

(5) $$Cell\;viability\;\%=\frac{Optical\;density\;of\;treated\;cells}{Optical\;density\;of\;control\;cells}\times 100$$

#### 2.5.4. THP-1 Differentiation with PMA

For macrophage-like (M0) differentiation, THP-1 cells (1 × 10^5^/mL; 2 mL/well in 6-well plates) were treated with the optimal PMA concentration (200 ng/mL) for 24 h, leading to adherence and spreading. PMA-containing medium was then removed, cells were washed once with PBS, and fresh medium was added for a 24 h resting period. Differentiation was confirmed by morphological changes, including adherence and flattening.

#### 2.5.5. Cytotoxicity and Apoptosis Evaluation of Nanoparticles Using MTT Assay and Annexin V–FITC/PI Flow Cytometry

The cytotoxicity of chitosan, piperine, non-targeted nanoparticles, and targeted nanoparticles was evaluated in THP-1 (1 × 10^4^ cells/well) and RAW 264.7 (8 × 10^3^ cells/well) cells using the MTT assay in 96-well plates. Cells were seeded and allowed to attach (RAW 264.7) or stabilize (THP-1) for 24 h prior to treatment. Subsequently, cells were exposed to various concentrations of nanoparticles (2–128 µg/mL) for 24 h. After treatment, 20 µL of MTT solution (0.5 mg/mL) was added to each well and incubated for 4 h at 37 °C. The resulting purple formazan crystals were dissolved in DMSO, and absorbance was measured at 570 nm using a microplate reader. Cell viability (%) was calculated using Equation (5), and results were expressed as mean ± SD (n = 3) [9].

To further investigate the mechanism of cytotoxicity, apoptosis was assessed using Annexin V–FITC/PI dual staining followed by flow cytometry analysis. THP-1 and RAW 264.7 cells were seeded in 6-well plates (3 × 10^5^ cells/well) and incubated for 24 h. Cells were then treated with selected concentrations of nanoparticles (64 and 128 µg/mL) for 24 h. After treatment, cells were harvested, washed twice with cold PBS, and resuspended in 1× Annexin V binding buffer. Subsequently, 5 µL of Annexin V–FITC and 5 µL of propidium iodide (PI) were added to each sample and incubated for 15 min at room temperature in the dark. After incubation, 400 µL of binding buffer was added, and samples were immediately analyzed by flow cytometry [62].

#### 2.5.6. Evaluation of Cellular Internalization of Nanoparticles

To assess nanoparticle internalization, THP-1 macrophages and RAW 264.7 cells were seeded on sterile coverslips in 24-well plates (1 × 10^5^ cells/well) and treated with free CM6, CM6-CTT-NPs, and CM6-MCTT-NPs (5 µg/mL) for 4 h. After washing three times with PBS to remove uninternalized material, cells were fixed with 4% paraformaldehyde and counterstained with DAPI (1 µg/mL) for 5 min. Coverslips were mounted with antifade medium and observed under a fluorescence microscope (Olympus, Tokyo, Japan; CM6: green, Ex/Em 450/505 nm; DAPI: blue, Ex/Em 358/461 nm) (Olympus, Japan; CM6: green, Ex/Em 450/505 nm; DAPI: blue, Ex/Em 358/461 nm). Uptake was evaluated qualitatively by cytoplasmic green fluorescence and quantitatively using Equation (6) to calculate Corrected Total Cell Fluorescence (CTCF) with ImageJ software (version 1.54g, NIH, Bethesda, MD, USA) [33,37,53].

(6) CTCF=Integrated Density−(Cell Area×Mean Background Fluorescence)

#### 2.5.7. Anti-Inflammatory Activity

##### Evaluation of Extracellular NO Production Using the Griess Method

To evaluate the effect of CTS, PIP, and their nanoparticle formulations on extracellular nitric oxide (NO) production in macrophages. RAW 264.7 and PMA-differentiated THP-1 cells were seeded in 24-well plates and incubated for 24 h at 37 °C in 5% CO_2_ with test samples (64 µg/mL), LPS (200 ng/mL; positive control), or vehicle control. After treatment, culture supernatants were collected and centrifuged at 500× *g* for 5 min to remove cell debris. Equal volumes of the supernatant and freshly prepared Griess reagent (1% sulfanilamide in 5% phosphoric acid mixed with 0.1% N-(1-naphthyl)ethylenediamine) were combined and incubated at room temperature for 10 min, protected from light. Absorbance was measured at 550 nm using a microplate reader (Synergy H1, BioTek Instruments, Winooski, VT, USA), and NO concentrations were calculated from NaNO_2_ standard curve. All measurements were performed in triplicate [63,64].

##### Evaluation of Intracellular NO Production Using DAF-FM DA

To assess intracellular nitric oxide (NO) levels, RAW 264.7 and PMA-differentiated THP-1 macrophages were seeded in 24-well plates containing sterile coverslips and allowed to adhere overnight. Cells were treated with CTS, PIP, non-targeted, and targeted nanoparticles (64 µg/mL), LPS (200 ng/mL; positive control), or vehicle control for 24 h at 37 °C in 5% CO_2_. Following treatment, cells were washed with PBS and incubated with 5 µM DAF-FM DA in serum-free medium for 30 min at 37 °C in the dark. Cells were additionally counterstained with DAPI (1 µg/mL) for 5 min to visualize nuclei. After probe loading, cells were rinsed with PBS and further incubated in fresh medium for 20 min to allow complete de-esterification of the probe. Intracellular NO-associated fluorescence was visualized using a Nikon AXR confocal laser scanning microscope (excitation/emission 495/515 nm) (Nikon, Tokyo, Japan). Fluorescence intensity was quantified using ImageJ software and the corrected CTCF was calculated according to Equation (6) [65].

##### Evaluation of Cytokine Gene Expression in Macrophages by qRT-PCR

To evaluate the anti-inflammatory potential of CTS, PIP, and their non-targeted and targeted nanoparticle formulations, THP-1 and RAW 264.7 cells were first activated with 200 ng/mL LPS for 24 h. Activated cells were treated with the test samples (64 µg/mL) for 24 h. Total RNA was isolated using TRIzol reagent, followed by the synthesis of complementary DNA (cDNA). Quantitative real-time PCR (qRT-PCR) was then conducted to determine the expression levels of pro-inflammatory cytokine genes, including TNF-α, IL-6, and IL-1β. Gene expression was normalized to β-actin, and relative changes were calculated using the 2^−ΔΔCt^ method. The primer sequences employed are provided in Table S1 [9].

#### 2.5.8. Antioxidant Activity

##### Radical Scavenging and Antioxidant Capacity Assays

The antioxidant activity of CTS, PIP, and their non-targeted and targeted nanoparticle formulations was assessed through DPPH, ABTS, FRAP, and TAC assays. Test samples were evaluated at concentrations ranging from 0.05 to 0.6 mg/mL, with all measurements carried out in triplicate. Radical scavenging and antioxidant capacities were expressed relative to the appropriate reference standards. A detailed description of the assay conditions, including reagents, incubation parameters, and detection wavelengths, is provided in Table 2.

(7) $$\%\;Scavenging=\frac{A_{control}{-A}_{test}}{A_{control}}\times 100$$

##### Assessment of Intracellular Reactive Oxygen and Nitrogen Species

To investigate the antioxidant effects of CTS, PIP, and their nanoparticle formulations, THP-1 and RAW 264.7 cells were incubated with the test samples at concentrations of 5–45 µg/mL for 30 min at 37 °C in 5% CO_2_. For ROS detection, cells were loaded with the ROS-sensitive dye 10 µM DCFH-DA in serum-free medium and incubated under dark conditions for 30 min to allow the probe to react with intracellular oxidants. The resulting fluorescence, reflecting ROS levels, was then recorded using a microplate reader (Agilent Synergy H1, BioTek Instruments, CA, USA) equipped for 488 nm excitation and 525 nm emission detection. Intracellular RNS levels were measured using the fluorogenic probe 10 µM BBoxiProbe™ R21F, (BestBio, Beijing, China) which reacts with reactive nitrogen species to generate a fluorescent signal. Fluorescence intensity from RNS detection was captured under similar conditions as ROS measurements. All assays were conducted in triplicate wells with three biological replicates, and the results were expressed relative to untreated control cells [70].

### 2.6. Statistical Analysis

Experimental results are reported as mean ± SD. Differences between groups were assessed using one-way or two-way ANOVA, depending on the experimental design. All statistical calculations and figure preparations were carried out using GraphPad Prism 10.4.2 (GraphPad Software, San Diego, CA, USA) and Origin 2018 (64-bit, OriginLab Corporation, Northampton, MA, USA).

## 3. Results and Discussion

### 3.1. Characterization of Chitosan–Mannose Conjugation and Stabilization with TPGS and Tween

#### 3.1.1. Functional Group Interactions and Piperine Encapsulation (FTIR)

**Figure 2:**
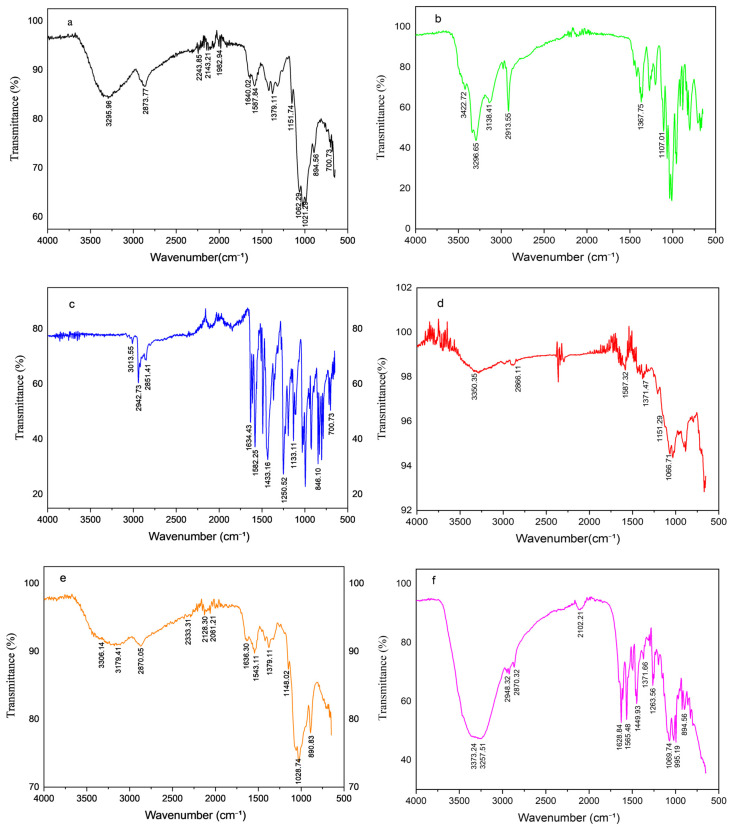
FTIR spectra of (**a**) chitosan, (**b**) mannose, (**c**) piperine, (**d**) physical mixture (PM-PIP-CTT), (**e**) PIP-CTT-NPs, and (**f**) PIP-MCTT-NPs.

FTIR spectroscopy was employed to confirm chemical interactions, functional group modifications, and the encapsulation of PIP within nanoparticle formulations. The FTIR spectra of CTS, MNS, PIP, the physical mixture (PM-PIP-CTT), and non-targeted and targeted nanoparticles are presented in Figure 2. CTS exhibited a broad absorption band at 3295 cm^−1^, corresponding to overlapping –OH and –NH_2_ stretching vibrations, along with a peak at 1587 cm^−1^ representing –NH bending (Figure 2a). MNS showed characteristic absorption peaks at 3422, 3296, 2913, 1367, and 1107 cm^−1^ (Figure 2b), consistent with carbohydrate hydroxyl and C–O–C stretching vibrations reported earlier, which served as reference signals for evaluating mannose conjugation [71]. PIP exhibited distinctive aromatic and amide-associated peaks (Figure 2c), confirming its structural identity. The PM-PIP-CTT exhibited superimposed characteristic peaks of CTS, MNS, and PIP without significant peak shifts or new peak formation, confirming simple physical blending of the components (Figure 2d).

In PIP-CTT-NPs, the spectra displayed a broad –OH/–NH stretching band at 3306 cm^−1^ and retention of piperine-associated signals, suggesting physical entrapment without covalent modification (Figure 2e). Following mannose functionalization, the –OH/–NH stretching band shifted to 3373 cm^−1^ with increased broadness, while additional changes appeared in the 1565 cm^−1^ (amine bending) region and a weak signal near 1450 cm^−1^ (Figure 2f). These variations indicate the formation of Schiff base (C=N) linkages between the aldehyde group of mannose and the primary amine groups of chitosan, along with hydrogen bonding between mannose hydroxyl groups and the hydroxyl/amine functionalities of chitosan [50]. In PIP-MCTT-NPs stabilized with TPGS and Tween 80, absorption features at 1100–1150 cm^−1^ supported the presence of polyether groups, confirming stabilizer incorporation. The broadening of the –OH/–NH region suggested hydrogen bonding between Tween 80 hydroxyl groups and chitosan functionalities, while PEG-related stretching confirmed successful TPGS incorporation [41,44]. Overall, FTIR confirmed mannose–chitosan interactions, stabilizer incorporation, and the physical entrapment of PIP while preserving its native structure.

#### 3.1.2. Nanoparticle Formation and Chromophore Stability (UV–Vis)

**Figure 3:**
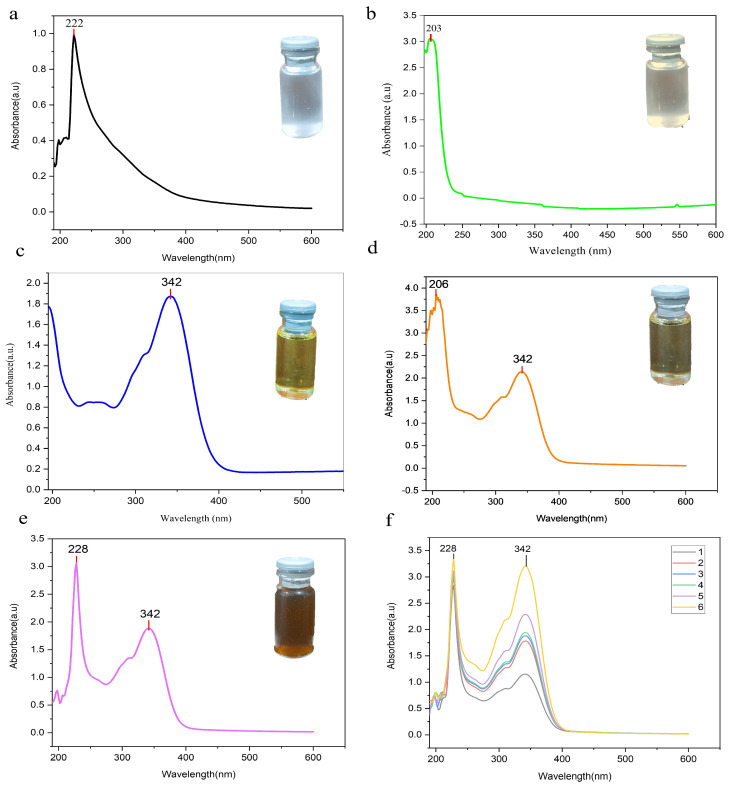
UV–vis spectra showing: (**a**) pure chitosan (foggy white) with a peak at 222 nm in 2% acetic acid, (**b**) mannose (chalky white) with a peak at 203 nm in acetate buffer, (**c**) piperine (light yellow) with a peak at 342 nm in ethanol, (**d**) PIP-CTT-NPs (moderate yellow) with peaks at 206 nm and 342 nm in 2% acetic acid, (**e**) PIP-MCTT-NPs (dark yellow) with peaks at 228 nm and 342 nm in 2% acetic acid, (**f**) PIP-MCTT-NPs at varying concentrations, consistently showing peaks at 228 nm and 342 nm.

UV–visible spectroscopy (200–600 nm) was employed as a complementary optical tool to verify nanoparticle formation and confirm drug encapsulation, similar to previous studies where UV–vis absorption and visible color changes were used to demonstrate nanoparticle formation and encapsulation efficiency [72]. CTS appeared foggy white with a peak at 222 nm (Figure 3a), while MNS had a chalky white appearance and a band at 203 nm (Figure 3b). PIP exhibited a light-yellow color with a distinct band at 342 nm, characteristic of its conjugated aromatic system (Figure 3c). Encapsulation into the chitosan–TPP matrix stabilized with TPGS and Tween 80 yielded moderately yellow PIP-CTT-NPs, showing peaks at 206 nm and 342 nm (Figure 3d). Mannose modification produced a darker-yellow suspension, with a minor shift in the shorter-wavelength peak to 228 nm while retaining the 342 nm piperine band (Figure 3e), indicating preservation of the chromophore. The absorbance remained stable across different concentrations of PIP-MCTT-NPs (Figure 3f), reflecting colloidal and stabilizer-mediated structural stability. These results agree with previous studies where chitosan nanoparticles showed color changes confirming curcumin encapsulation [55].

#### 3.1.3. Crystallinity Changes and Mannose Functionalization (XRD)

**Figure 4:**
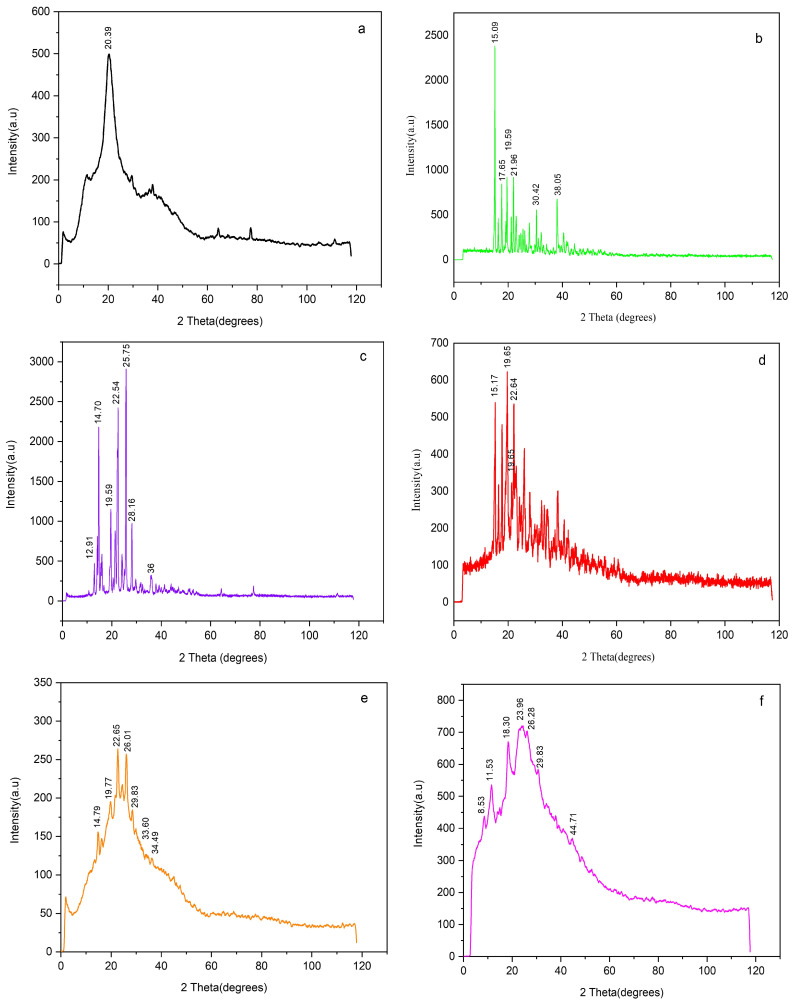
X-ray diffraction patterns of (**a**) chitosan, (**b**) mannose, (**c**) piperine, (**d**) physical mixture (PM-PIP-CTT), (**e**) PIP-CTT-NPs, and (**f**) PIP-MCTT-NPs.

XRD patterns revealed structural changes upon nanoparticle formulation (Figure 4). CTS exhibited a broad peak at 20.39°, indicative of its semi-crystalline nature, with a CrI of 60% (Figure 4a) [29]. MNS showed sharp peaks at 15.09°, 19.59°, and 21.96°, reflecting its crystalline structure (Figure 4b). PIP displayed sharp peaks at 12.91°, 14.70°, 19.59°, and 22.54°, consistent with its monoclinic crystalline form (JCPDS card no: 00-043-1627) (Figure 4c) [73]. The physical mixture (PM-PIP-CTT) retained the characteristic crystalline peaks of PIP and MNS at 15.17°, 19.65°, and 22.64°, along with the broad semi-crystalline halo of chitosan around 20°, without significant peak disappearance or major shifts, confirming simple physical blending without structural transformation or amorphization (Figure 4d).

After nanoparticle formation, PIP-CTT-NPs exhibited broader peaks at 19.77° and 22.65°, with CrI reduced to 33%, indicating partial amorphization and structural reorganization of the polymer matrix (Figure 4e). PIP-MCTT-NPs, stabilized with TPGS and Tween 80, showed peaks at 8.53°, 11.53°, and 18.30°, with CrI further reduced to 30%, suggesting additional rearrangements due to mannose functionalization and stabilizer incorporation (Figure 4f). The reduced intensity and broadening of PIP peaks in both nanoparticle formulations confirm partial amorphization and successful encapsulation. These XRD results complement FTIR and UV–vis findings, supporting polymer–drug and mannose–chitosan interactions, as well as stabilizer effects, while serving as supportive evidence of nanoparticle formation. These results agree with previous studies [74].

#### 3.1.4. Elemental Composition and Mannose Conjugation (CHNS)

Elemental analysis was performed to determine the chemical composition of CTS, MNS, PIP, and their nanoparticle formulations, and to confirm mannose conjugation in PIP-MCTT-NPs (Table 3). CTS showed 48.18% C, 8.12% H, 7.65% N, and 38.72% O, consistent with literature [50]. MNS exhibited high hydrogen content (12.55%) and low nitrogen (0.02%), characteristic of carbohydrates. PIP displayed high carbon content (71.56%), reflecting its aromatic structure. PIP-CTT-NPs retained elemental profiles similar to CTS and PIP, indicating successful encapsulation without chemical modification. PIP-MCTT-NPs, stabilized with TPGS and Tween 80, showed decreased nitrogen (3.22%) and increased oxygen (44.08%), confirming mannose conjugation, with a calculated degree of substitution (DS) of 50.73%. These findings align with FTIR and XRD data, confirming mannose conjugation, stabilizer presence, and maintenance of PIP integrity within nanoparticles. Overall, these results are consistent with previous studies where chitosan nanoparticles incorporated bioactive compounds while retaining their structural characteristics [50,71].

#### 3.1.5. DLS Analysis

DLS analysis was used to evaluate the mean particle size, polydispersity index (PDI), and zeta potential of PIP- and CM6-loaded nanoparticles (Table 4). The average particle sizes of PIP-CTT-NPs, PIP-CMTT-NPs, CM6-CTT-NPs, and CM6-CMTT-NPs were 78.02 ± 1.20 nm, 162.65 ± 0.66 nm, 76.07 ± 0.29 nm, and 160.18 ± 1.93 nm, respectively. These values place all formulations within the nanometer range, which is generally considered suitable for drug delivery and cellular uptake [75]. A noticeable increase in particle size was observed after mannose conjugation, consistent with previous studies where surface functionalization increased hydrodynamic diameter [37]. The PDI values ranged between 0.196 and 0.288, indicating relatively narrow size distributions, with all values remaining below the 0.3 threshold typically associated with monodisperse nanoparticle systems [50,76]. The zeta potential values demonstrated a positive surface charge in all formulations, as expected for chitosan-based nanoparticles. Notably, non-targeted nanoparticles exhibited higher zeta potentials compared to mannose-modified ones, which may be attributed to partial neutralization of surface amino groups following mannose conjugation [33]. The replacement of 2.5 mg piperine with 0.2 mg coumarin-6 did not markedly alter particle size or PDI, likely because the overall architecture of the nanoparticles is governed primarily by the chitosan–TPP network. Since coumarin-6 is used at much lower concentrations and is hydrophobic in nature, its incorporation had only a minimal effect on nanoparticle dimensions.

#### 3.1.6. EE% and DL%

The drug loading and encapsulation efficiency of PIP and CM6 were quantitatively assessed for both non-targeted and mannose-conjugated targeted nanoparticles using standard calibration curves. PIP-CTT-NPs exhibited a drug loading capacity of 2.31 mg, whereas PIP-MCTT-NPs showed a slightly reduced value of 2.05 mg. Similarly, CM6-CTT-NPs and CM6-MCTT-NPs displayed drug loadings of 2.30 mg and 2.00 mg, respectively. Encapsulation efficiency was determined using the indirect method, wherein the unentrapped drug was quantified from the supernatant after centrifugation. PIP-CTT-NPs achieved a high EE of 92.48 ± 1.56%, while PIP-MCTT-NPs showed a slightly lower EE of 82.32 ± 2.79%. A comparable trend was observed for CM6-loaded nanoparticles, with CM6-CTT-NPs demonstrating 92.18 ± 2.73% and CM6-MCTT-NPs yielding 80.25 ± 1.62% (Table 4). The modest reduction in EE and DL in the mannose-conjugated formulations may be attributed to surface modification, as mannose attachment could alter nanoparticle surface characteristics and partially hinder drug entrapment during the ionic cross-linking and emulsification steps [50]. Nevertheless, all formulations retained high encapsulation efficiencies, indicating effective drug incorporation with minimal loss during preparation. These findings confirm that mannose-modified nanoparticles, stabilized with TPGS and Tween 80, are stable and possess functionalized surfaces and sizes appropriate for targeted drug delivery. Alexis et al. 2008 [77] stated that polymeric nanoparticles can significantly influence the pharmacokinetics and biodistribution of encapsulated drugs, thereby enhancing therapeutic outcomes in vivo, with these effects largely determined by particle size, surface properties, and functionalization strategies. Similarly, Pratten et al. 1984 [78] reported that nanoparticle size plays a critical role in cellular uptake, as smaller particles are more effectively internalized by Kupffer cells and other phagocytes of the reticuloendothelial system, thereby influencing their biological fate.

#### 3.1.7. Stability

Stability assessment revealed only a slight increase in particle size for PIP-CTT-NPs (2.6%) and PIP-MCTT-NPs (1.3%) after storage, confirming good stability of both formulations. Importantly, zeta potential and PDI remained nearly unchanged, indicating preservation of surface charge and uniform size distribution (Table 5). These findings demonstrate that the colloidal stability of the nanoparticles was well maintained over time. Lyophilization further enhanced structural and physicochemical stability by reducing residual moisture and limiting molecular mobility. Despite the absence of a cryoprotectant, no significant aggregation or structural destabilization was observed, as evidenced by minimal post-lyophilization changes in particle size, PDI, and zeta potential. The ionic cross-linked chitosan–TPP matrix, together with steric stabilization provided by TPGS and Tween 80, likely contributed to maintaining nanoparticle integrity during freeze-drying.

Similar observations have been reported previously, where freeze-drying was shown to preserve nanoparticle size, surface charge, and encapsulation efficiency during long-term storage [32,37]. Overall, these results validate the robustness and formulation stability of the PIP-CTT-NP and PIP-MCTT-NP systems, underscoring their suitability for sustained drug delivery and therapeutic applications.

#### 3.1.8. SEM Analysis

**Figure 5:**
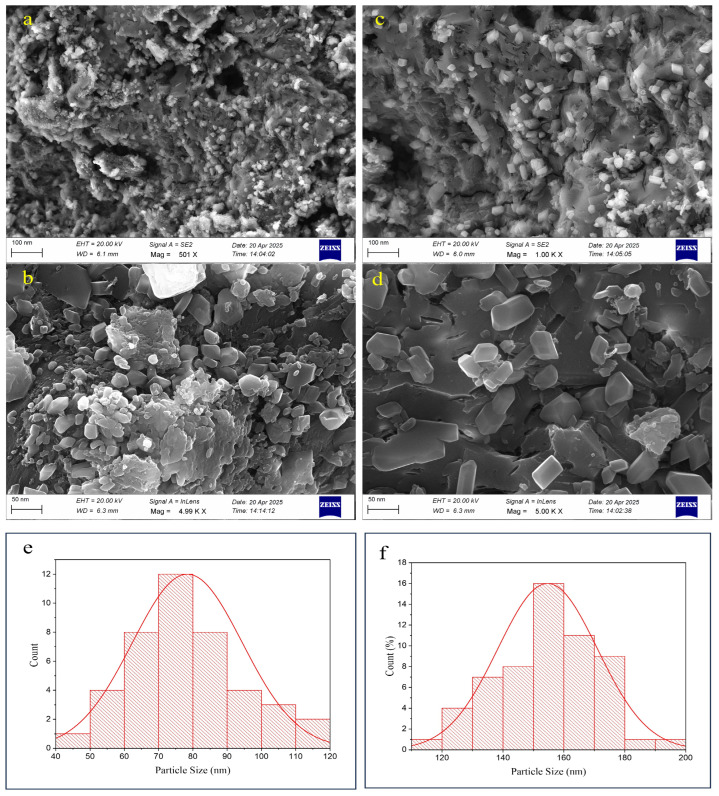
SEM images and particle size distribution histograms of the synthesized nanoparticles. (**a**,**b**) SEM micrographs of PIP-CTT-NPs, (**c**,**d**) SEM images of PIP-MCTT-NPs, (**e**) particle size distribution of PIP-CTT-NPs, (**f**) particle size distribution of PIP-MCTT-NPs.

SEM was employed to examine the surface morphology and structural features of the synthesized nanoparticles (Figure 5). Both formulations exhibited irregular cubic to slightly spherical morphologies, with occasional rod-shaped structures. The nanoparticle surfaces appeared moderately textured without obvious defects such as pinholes or cracks, which is in line with previous observations for chitosan-based nanoparticles prepared via ionic gelation [79]. A visual comparison between PIP-CTT-NPs (Figure 5a,b) and PIP-MCTT-NPs (Figure 5c,d) indicated a relative increase in particle dimensions following mannose modification. To provide quantitative insight, particle size distributions were obtained by analyzing SEM images (n = 50) using image analysis software (Figure 5e,f). The mean particle sizes were estimated to be 78.37 nm for PIP-CTT-NPs and 154.66 nm for PIP-MCTT-NPs. These values were lower than those determined by DLS, which is expected since SEM captures dehydrated, solid-state dimensions, whereas DLS measures hydrodynamic diameter in aqueous suspension. The observed increase in size upon mannose modification was consistent with the DLS findings and with previous reports indicating that surface functionalization can alter nanoparticle dimensions [33]. SEM provided morphological confirmation and supported the size trends observed by DLS; however, it should be considered complementary to scattering-based methods for precise nanoparticle size determination.

#### 3.1.9. TEM Analysis

**Figure 6:**
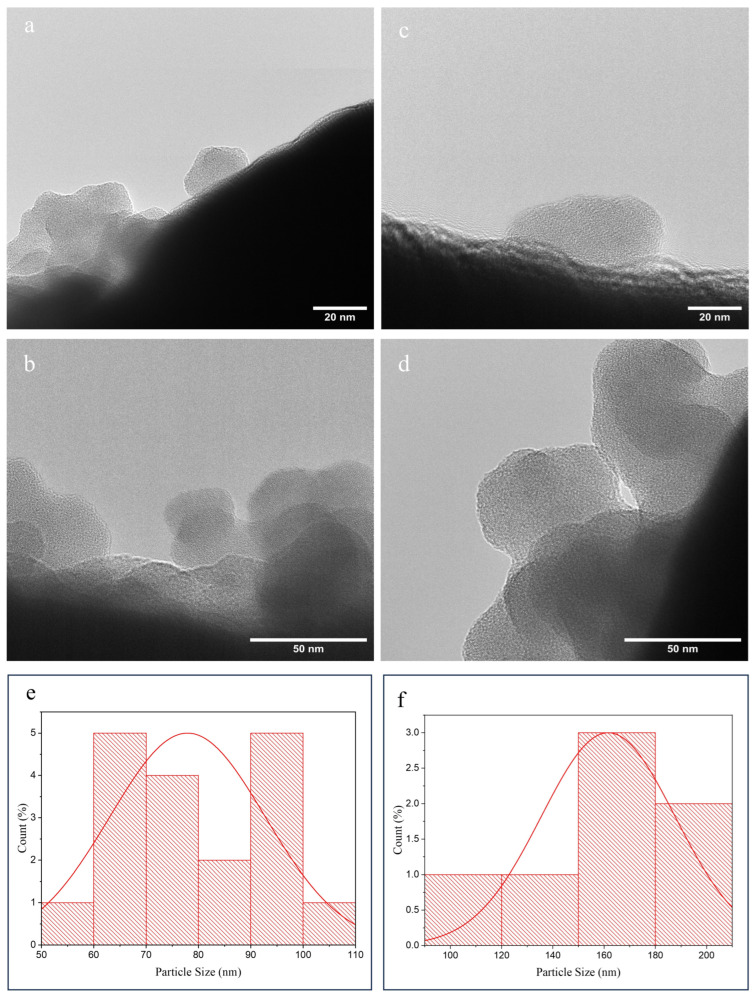
TEM images and particle size distribution histograms of the synthesized nanoparticles. (**a**,**b**) TEM micrographs of PIP-CTT-NPs, (**c**,**d**) TEM images of PIP-MCTT-NPs, (**e**) Particle size distribution histogram of PIP-CTT-NPs, (**f**) Particle size distribution histogram of PIP-MCTT-NPs based on measurements from TEM image analysis.

TEM was employed to evaluate the morphology and size of nanoparticles (Figure 6). Images revealed that both PIP-CTT-NPs and PIP-MCTT-NPs exhibited irregular cubic to slightly spherical shapes (Figure 6a–d). Quantitative analysis of 15 particles per formulation estimated mean sizes of 77.91 nm for PIP-CTT-NPs and 161.59 nm for PIP-MCTT-NPs (Figure 6e,f), consistent with SEM observations and following the size trend observed by DLS. The slightly smaller dimensions compared to DLS measurements are expected, as TEM captures dehydrated, solid-state particles, whereas DLS measures hydrodynamic diameters in dispersion [80]. These complementary imaging techniques confirm the nanoscale morphology and provide higher-resolution validation of particle size and shape, supporting the structural integrity of the formulations and the effects of mannose functionalization on nanoparticle dimensions.

#### 3.1.10. AFM Analysis

**Figure 7:**
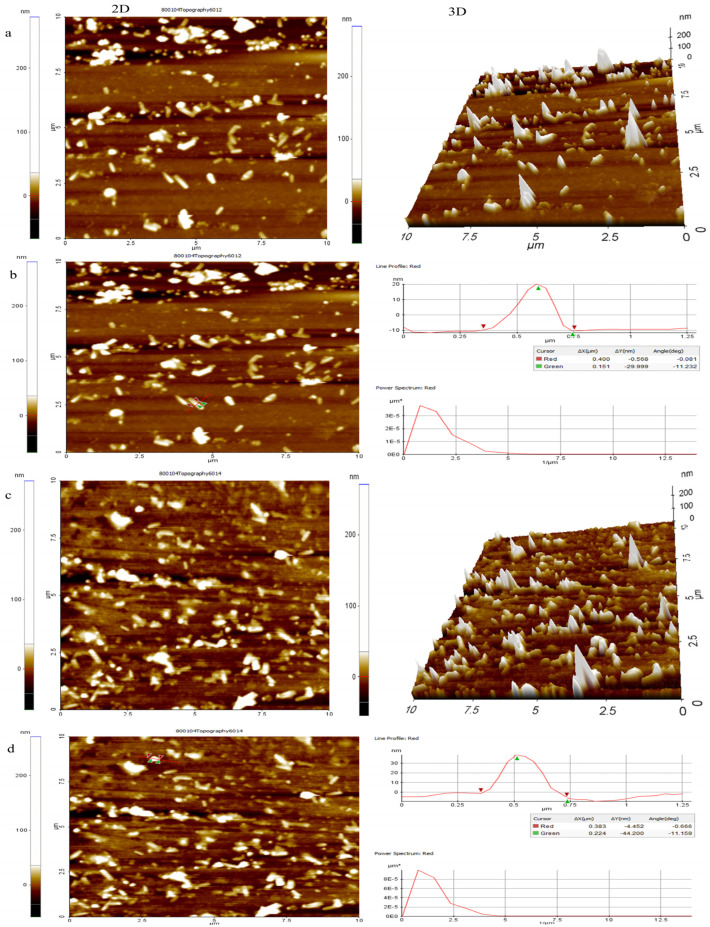
AFM analysis of nanoparticles. (**a**) 2D and 3D topographical images of PIP-CTT-NPs, (**b**) Surface roughness profile of PIP-CTT-NPs, (**c**) 2D and 3D topographical images of PIP-MCTT-NPs, (**d**) Surface roughness profile of PIP-MCTT-NPs.

AFM was employed to assess the surface morphology and roughness of PIP-CTT-NPs and PIP-MCTT-NPs (Figure 7a–d). PIP-CTT-NPs showed height values ranging from −12 to 20 nm, with a mean height of 3.2 nm and a peak-to-valley roughness (RPV) of 32 nm. Mannose-conjugated PIP-MCTT-NPs exhibited increased height variations (−9 to 38 nm), a mean height of 4.6 nm, and RPV of 44 nm, indicating greater surface irregularity. Surface roughness parameters further revealed Ra/Rq values of 6.4/8.8 nm for PIP-CTT-NPs and 9.8/12.4 nm for PIP-MCTT-NPs, with negative skewness and leptokurtic distributions, highlighting valley-dominated, sharply featured surfaces. These results are consistent with the observations obtained from SEM, TEM, and DLS analyses. The lower particle density observed in TEM and AFM images is attributed to the localized and surface-based nature of these techniques, which analyze a limited number of particles under dehydrated conditions. In contrast, DLS provides an ensemble measurement of particles in a fully hydrated dispersion, representing the overall particle population. Additionally, the reduced visibility of nanoparticles in TEM and AFM may result from low sample concentration, non-uniform deposition on the substrate, and the inherently low electron density of polymeric nanoparticles. Therefore, this observation does not indicate the absence of nanoparticles but rather reflects methodological limitations. Furthermore, the increased surface roughness observed in mannose-modified nanoparticles may enhance cellular interactions and uptake, supporting their potential as targeted drug delivery carriers [81,82].

### 3.2. In Vitro Studies

#### 3.2.1. In Vitro Release Studies of Piperine from PIP-CTT-NPs and PIP-MCTT-NPs

**Figure 8:**
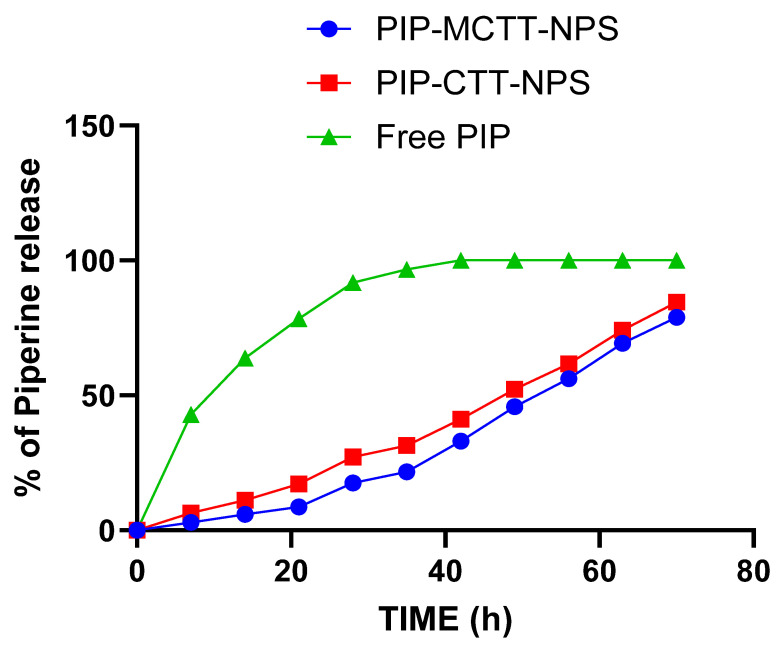
In vitro release profile of PIP from PIP-CTT-NPs and PIP-MCTT-NPs in phosphate-buffered saline (PBS, pH 7.4) over 72 h. Data are presented as mean ± SD (n = 3 independent experiments). The standard deviation values are very small due to the high reproducibility of the formulations, and therefore error bars may not be clearly visible. For comparison, free piperine exhibited rapid diffusion-controlled release, with >90% release within 6–8 h under identical experimental conditions.

The in vitro release of PIP was evaluated under physiological conditions (PBS, pH 7.4) to assess the sustained delivery potential of the developed nanoparticle systems (Figure 8). Both non-targeted and mannose-targeted formulations exhibited controlled release profiles over a period exceeding 36 h, with no evidence of an initial burst release. At 72 h, cumulative PIP release reached 84.3% for PIP-CTT-NPs and 78.7% for PIP-MCTT-NPs. The slightly lower release observed for the mannose-modified formulation is likely attributable to surface functionalization, which introduces additional diffusion barriers and modulates drug release kinetics [83]. The absence of burst release and the prolonged release behavior can be ascribed to the combined stabilizing effects of TPGS and Tween 80 within the nanoparticle matrix, providing improved control over drug diffusion compared with conventional delivery systems. Consistent with these findings, previous studies have reported that TPGS-based carriers enhance the sustained release and solubility of poorly water-soluble drugs [33], while Tween 80-stabilized nanoparticles exhibit improved colloidal stability and controlled drug release characteristics [40,41].

For comparison, free piperine exhibited rapid diffusion-controlled release under identical experimental conditions, with cumulative release values of 42% at 1 h, 63% at 2 h, 78% at 4 h, 91% at 6 h, 96% at 8 h, and complete release (100%) within 12 h. This rapid release behavior reflects the absence of polymeric diffusion barriers and is consistent with the poor aqueous solubility of piperine, which results in an initial burst release when evaluated in dialysis-based in vitro systems. The pronounced difference between free piperine and nanoparticle-encapsulated formulations clearly demonstrates the ability of the chitosan–TPGS–Tween 80 matrix to effectively regulate drug diffusion and prolong release duration.

In comparison with previously reported formulations, the present nanoparticle systems demonstrated superior controlled release behavior. Darwish et al. reported piperine-loaded chitosan lipid nanoparticles (PP-CLNPs) exhibiting a pronounced burst release within the first 6 h, followed by sustained release, with cumulative release values ranging from 86% to 99% at 24 h [74]. Similarly, Gilani et al. developed piperine-loaded polycaprolactone nanoparticles (PPN-PCL-NPs) that showed an initial burst release of 47.82% within 6 h [84]. In contrast, both PIP-CTT-NPs and PIP-MCTT-NPs achieved sustained release without an initial burst effect, highlighting the superior stabilizing influence of the TPGS–Tween 80 combination in regulating drug release kinetics.

Although these in vitro release studies represent baseline release behavior, the release kinetics of chitosan-based nanocarriers are expected to be influenced by physiological microenvironments. Mildly acidic conditions present in inflamed tissues and endosomal compartments can enhance chitosan protonation, swelling, and matrix relaxation, potentially accelerating piperine release. Furthermore, intracellular enzyme-rich environments may promote chitosan degradation, thereby facilitating drug liberation. Accordingly, piperine release from the developed nanocarriers is anticipated to be more pronounced under such conditions, supporting their suitability for intracellular and inflammation-targeted delivery.

From a translational perspective, the observed 48–72 h sustained release profile without an initial burst is particularly suited for inflammation-driven and macrophage-mediated applications, where controlled short-term intracellular drug availability is therapeutically relevant, such as acute inflammatory responses, intracellular bacterial infections, and repeated-dose nanotherapeutic interventions. In contrast, chronic degenerative conditions such as osteoarthritis may require delivery systems capable of maintaining therapeutic drug levels over extended periods (weeks). The present formulation was therefore designed to prioritize controlled, burst-free release and efficient cellular targeting rather than long-term depot delivery. Further formulation modifications would be required to achieve extended-release profiles, which were beyond the scope of the current study.

#### 3.2.2. Cytotoxicity Assessment of PMA in THP-1 Cells

To confirm the safe concentration range of PMA for THP-1 cells, we examined its effects on cell proliferation across different doses. As shown in Figure S2, PMA concentrations below 200 ng/mL did not cause any significant reduction in cell proliferation when compared to the control group (0 ng/mL). However, higher concentrations of 300 and 400 ng/mL induced a marked decline in cell viability, with statistically significant reductions observed (*p* < 0.0001). These findings indicate that PMA concentrations above 200 ng/mL are cytotoxic to THP-1 cells, whereas concentrations below this threshold are relatively safe. This observation is consistent with earlier findings by Liu et al., 2023 [61], who also reported no significant effects on proliferation below 200 ng/mL, but clear cytotoxicity at higher doses.

#### 3.2.3. PMA-Induced Differentiation of THP-1 Cells into Macrophages

As shown in Figure S3, untreated THP-1 cells retained their proliferative morphology over time, while PMA-treated cells displayed a distinct shift toward a macrophage-like phenotype. Using the non-cytotoxic concentration of 200 ng/mL (as established in Figure S2), cells exhibited adherence, flattening, and a cessation of proliferation after 24 h, characteristic of non-polarized (M0) macrophages. These morphological changes, indicated by yellow arrows, confirm successful differentiation and are consistent with previous reports [85].

#### 3.2.4. Cytotoxicity and Apoptosis of Nanoparticle Formulations in Macrophages

##### Cytotoxicity of Nanoparticle Formulations in Macrophages

The cytotoxic effects of CTS, PIP, and their nanoparticle formulations were evaluated in RAW 264.7 and THP-1 macrophages using the MTT assay. As shown in Figure S4, concentrations up to 64 μg/mL exhibited negligible effects on cell viability in both cell types. At higher concentrations, a significant, dose-dependent decrease in viability was observed, particularly for the nanoparticle formulations. At 128 μg/mL, PIP-CTT-NPs and PIP-MCTT-NPs reduced cell viability by 43.7% and 55.7% in RAW 264.7 cells (Figure S4A) and by 47.1% and 50.7% in THP-1 cells (Figure S4B), respectively. In contrast, free PIP and CTS at the same dose reduced viability by ≤19.5%. The cytotoxicity profile followed the order: PIP-MCTT-NPs > PIP-CTT-NPs > PIP > CTS.

The greater cytotoxic effect of PIP-MCTT-NPs can be attributed to mannose receptor–mediated uptake, which facilitates enhanced internalization into macrophages [38,53]. Furthermore, the improved activity of both nanoparticle formulations relative to free PIP likely results from their increased solubility and sustained release behavior, supported by stabilization with TPGS and Tween 80. These observations are consistent with earlier reports indicating that piperine nanoparticles display higher cellular cytotoxicity than free piperine due to improved uptake efficiency and controlled release [33,46,86]. Duan et al. (2023) demonstrated that free piperine in RAW 264.7 cells exhibited no cytotoxicity below 40 μg/mL, maintaining > 99% viability [87], whereas nano-encapsulated piperine showed a clear dose-dependent reduction in viability.

Similarly, Hatami et al. (2022) reported that mannose-decorated nanoparticles maintained > 95% viability in U937 macrophages at concentrations up to 200 μg/mL, confirming their excellent biocompatibility [38]. The enhanced cytotoxic response of PIP-MCTT-NPs compared to free PIP can therefore be attributed to mannose receptor–mediated endocytosis, which promotes preferential nanoparticle uptake in macrophages. Based on these findings, concentrations ≤ 64 μg/mL, which maintained > 80% viability in both RAW 264.7 and THP-1 cells, were considered non-cytotoxic and thus selected for subsequent anti-inflammatory and antioxidant assays.

##### Apoptosis Analysis of Nanoparticle-Treated Macrophages

To determine whether the reduction in cell viability observed in the MTT assay was associated with apoptosis induction, Annexin V–FITC/PI staining followed by flow cytometry was performed.

At 64 μg/mL, both RAW 264.7 and THP-1 macrophages maintained high viability, with control groups showing approximately 94–95% viable cells. Free PIP maintained 87.46 ± 0.31% (RAW 264.7) and 84.72 ± 0.57% (THP-1) viable cells. PIP-CTT-NPs and PIP-MCTT-NPs also maintained relatively high viability in RAW 264.7 cells (>84%), while THP-1 cells exhibited slightly greater sensitivity, particularly with PIP-MCTT-NPs (72.78 ± 1.37%).

**Figure 9:**
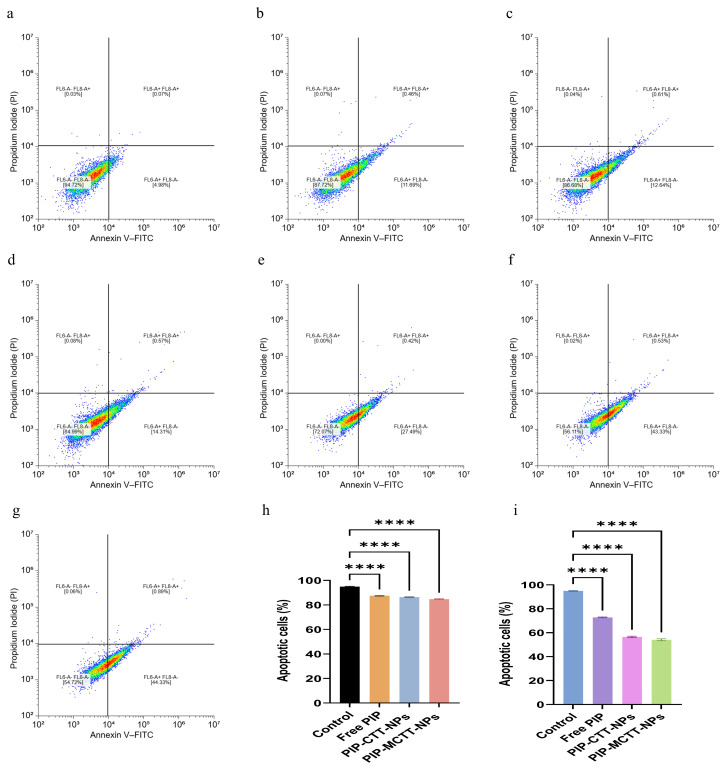
Annexin V–FITC/PI apoptosis analysis of RAW 264.7 macrophages treated with free PIP and nanoparticle formulations. Representative flow cytometry dot plots are shown for (**a**) untreated control cells; (**b**–**d**) cells treated with free PIP, PIP-CTT-NPs, and PIP-MCTT-NPs, respectively, at 64 μg/mL; and (**e**–**g**) cells treated with free PIP, PIP-CTT-NPs, and PIP-MCTT-NPs, respectively, at 128 μg/mL. Quantitative analysis of total apoptotic cells (early + late apoptosis) is presented in (**h**) for 64 μg/mL and (**i**) for 128 μg/mL. Data are expressed as mean ± SD (n = 3 independent experiments). **** indicates *p* < 0.0001; colors represent the respective cell populations.

At 128 μg/mL, a pronounced reduction in viable cell percentage was observed, consistent with the MTT results. In RAW 264.7 cells, viability decreased to 72.79 ± 0.35% for free PIP, 56.48 ± 0.59% for PIP-CTT-NPs, and 54.23 ± 0.84% for PIP-MCTT-NPs. Similarly, THP-1 cells exhibited 68.15 ± 0.79%, 55.08 ± 0.67%, and 53.14 ± 0.80% viability for free PIP, PIP-CTT-NPs, and PIP-MCTT-NPs, respectively (Figure 9, Raw; Figure S5, THP-1).

Importantly, quadrant analysis demonstrated that the decrease in viable cells at higher concentrations was primarily associated with increased early and late apoptotic populations rather than necrosis, indicating that nanoparticle-induced cytotoxicity occurred predominantly through apoptosis-mediated mechanisms, as determined by Annexin V–FITC/PI dual staining, a well-established method for distinguishing between viable, early apoptotic, late apoptotic, and necrotic cells [88]. The apoptotic trend followed the same order observed in the MTT assay: PIP-MCTT-NPs > PIP-CTT-NPs > free PIP, confirming that enhanced cellular uptake of mannose-functionalized nanoparticles contributes to increased apoptosis induction in macrophages through receptor-mediated endocytosis [89,90].

#### 3.2.5. Evaluation of Nanoparticle Uptake in Macrophages

**Figure 10:**
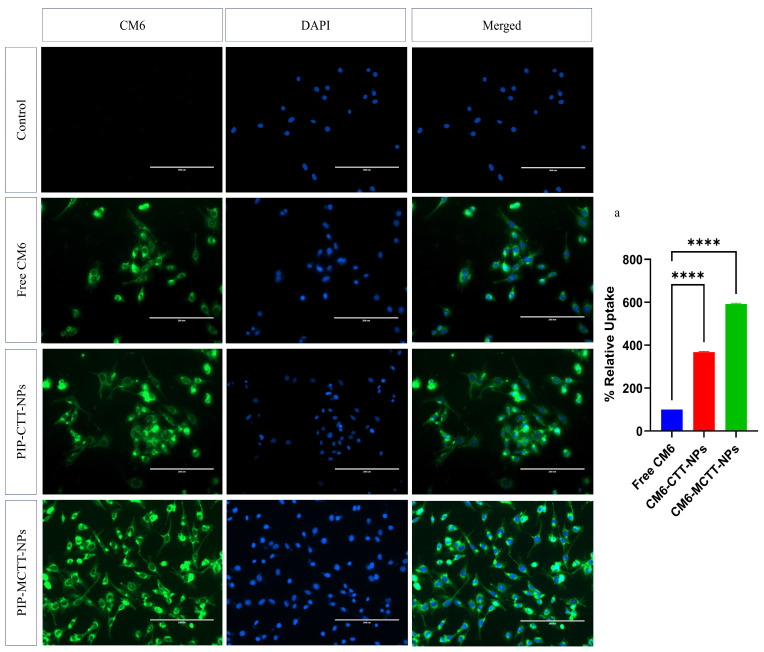
Fluorescence microscopy images of RAW 264.7 macrophages after 4 h incubation with control (no CM6), free CM6, CM6-CTT-NPs, and CM6-MCTT-NPs. Scale bar = 200 µm. (**a**) Quantitative analysis of relative cellular uptake expressed as percentage (%) relative to free CM6 (set as 100%). Data are presented as mean ± SD (n = 3 independent biological experiments). **** indicates *p* < 0.0001 compared to control.

**Figure 11:**
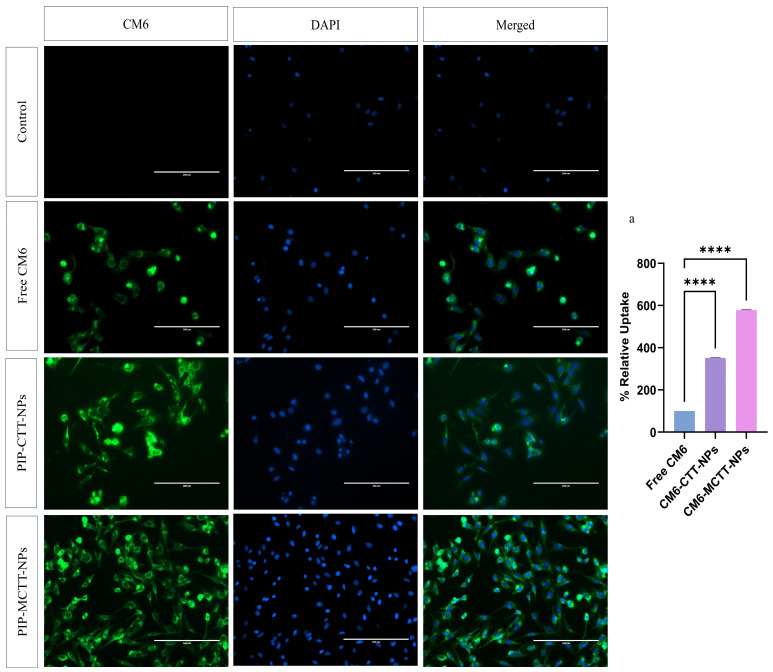
Fluorescence microscopy images of THP-1 macrophages after 4 h incubation with control (no CM6), free CM6, CM6-CTT-NPs, and CM6-MCTT-NPs. Scale bar = 200 µm. (**a**) Quantitative analysis of relative cellular uptake expressed as percentage (%) relative to free CM6 (set as 100%). Data are presented as mean ± SD (n = 3 independent biological experiments). **** indicates *p* < 0.0001 compared to control.

Since PIP is non-fluorescent, CM6, a highly sensitive fluorescent dye, was employed to label nanoparticles for cellular uptake studies [45]. RAW 264.7 and THP-1 macrophages were incubated with free CM6, CM6-CTT-NPs, and CM6-MCTT-NPs (equivalent to 5 µg/mL CM6), followed by nuclear staining with DAPI. Fluorescence microscopy using a FITC filter revealed no green fluorescence in the negative control (cells without CM6), confirming the absence of background signal. Cells treated with free CM6 displayed minimal cytoplasmic fluorescence (100% relative uptake), reflecting poor internalization due to limited solubility [33]. The CM6-CTT-NPs showed moderate fluorescence, corresponding to 368–351% relative uptake, whereas CM6-MCTT-NPs produced markedly higher fluorescence, achieving 591–577% relative uptake in both cell types, indicating superior internalization (Figure 10 and Figure 11). Normalized CTCF values per DAPI-stained nucleus (Table S2) confirmed 5.8–5.9× greater uptake of CM6-MCTT-NPs versus free CM6 across both cell lines, eliminating field-to-field cell number variation bias. The enhanced uptake of CM6-labeled nanoparticles can be attributed to the stabilizing and solubilizing effects of TPGS and Tween 80, which maintain nanoparticle integrity, prevent aggregation, and facilitate efficient intracellular delivery [33,46,86]. Furthermore, mannose functionalization promotes receptor-mediated endocytosis and bioadhesion, further increasing the uptake of CM6-MCTT-NPs compared to CM6-CTT-NPs [38]. The nanoparticle size (100–200 nm) is also optimal for phagocytic internalization, consistent with reports that macrophages preferentially internalize nanoparticles within this size range [78]. Collectively, these results demonstrate that surfactant-stabilized, mannose-functionalized nanoparticles markedly enhance cellular internalization and bioavailability compared with free CM6 or non-targeted nanoparticles. The findings indicate that TPGS/Tween 80-stabilized, mannose-functionalized chitosan nanoparticles can increase macrophage uptake by nearly six-fold, highlighting their potential as an efficient nanocarrier platform for targeted intracellular delivery of piperine and other bioactive compounds.

These results compare favorably with previous studies on mannose-targeted nanoparticle systems. Hatami et al. demonstrated that mannose-decorated hybrid nanoparticles (MDNPs) showed dose-dependent uptake by both M1 and M2 macrophages, with significantly higher uptake in M2 phenotype macrophages [38]. Similarly, Luan et al. reported that mannosamine-engineered PLGA-PEG nanoparticles achieved four-fold higher macrophage uptake rates compared to non-targeted PLGA-PEG systems at 24 h, mediated through mannose receptor-dependent endocytosis [91]. In contrast, Hoppstädter et al. found that M2-polarized macrophages internalized silica nanoparticles more efficiently than M1 cells, with significantly higher geometric mean fluorescence intensity values [92]. The six-fold enhancement in uptake observed with our PIP-MCTT-NPs represents a substantial improvement over conventional targeting strategies, likely due to the synergistic effects of mannose functionalization and TPGS/Tween 80 stabilization, enhancing both receptor-mediated endocytosis and nanoparticle solubilization.

It should be noted that fluorescent labeling was employed to provide qualitative insight into macrophage uptake behavior. Although CM6-loaded nanoparticles exhibited lower zeta potential than the piperine-loaded formulations, the uptake analysis was performed comparatively under identical formulation conditions. Direct intracellular quantification of piperine or labeling strategies that preserve nanoparticle surface charge would further strengthen uptake validation.

#### 3.2.6. Anti-Inflammatory Evaluation

##### Extracellular NO Inhibition

**Figure 12:**
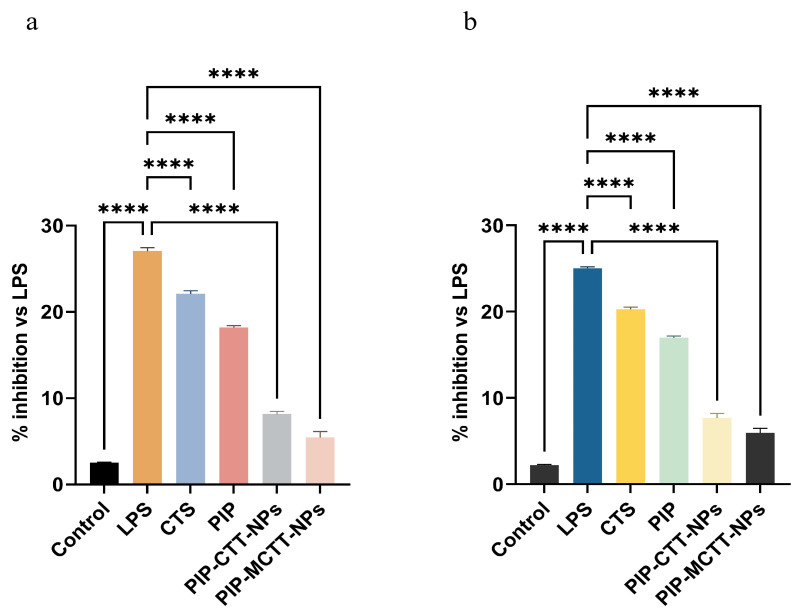
Effect of CTS, PIP, PIP-CTT-NPs, and PIP-MCTT-NPs on extracellular nitric oxide (NO) production in (**a**) RAW 264.7 and (**b**) THP-1 macrophages following LPS stimulation. Data are presented as mean ± SD (n = 3 independent biological experiments). Statistical analysis was performed using one-way ANOVA (*p* < 0.0001). **** indicates *p* < 0.0001 compared to control.

As shown in Figure 12, treatment with LPS robustly elevated NO production in both RAW 264.7 and THP-1 macrophages, confirming successful induction of an inflammatory response [93]. CTS and PIP moderately reduced NO levels, indicating some anti-inflammatory effect, but the reduction was significantly enhanced when PIP was delivered via nanoparticles. The non-targeted PIP-CTT-NPs reduced extracellular NO more effectively than free PIP, suggesting that the TPGS- and Tween 80-stabilized chitosan nanoparticles improve PIP solubility and cellular uptake, allowing greater intracellular delivery and subsequent suppression of inflammatory signaling [33,40]. The mannose-functionalized PIP-MCTT-NPs exhibited the highest inhibition of NO, reflecting targeted uptake via mannose receptors on macrophages, which concentrates the nanoparticles inside the cells and enhances the anti-inflammatory effect [94]. These results demonstrate a formulation-dependent effect, where TPGS and Tween 80 stabilize the nanoparticles and enhance dispersibility, while mannose targeting promotes cellular internalization. The combination of these factors results in a more potent suppression of NO production compared to free PIP or non-targeted nanoparticles.

Previous studies reported that TPGS and Tween 80 potentiate anti-inflammatory responses by enhancing solubility, bioavailability, and intracellular transport of encapsulated molecules [33,40,42,47]. Duan et al. showed that free piperine at 20 μg/mL achieved 39.13% reduction in NO secretion in LPS-stimulated RAW 264.7 cells [87]. In comparison, our mannose-functionalized PIP-MCTT-NPs demonstrated superior NO inhibition at lower concentrations, highlighting the enhanced bioavailability and targeted delivery of this formulation. Similarly, Bae et al. found that piperine inhibited LPS-induced TNF-α production in peritoneal macrophages, and the improvement achieved through nanoparticle delivery and mannose targeting represents a significant advancement in therapeutic efficacy [95].

##### Intracellular NO Inhibition

**Figure 13:**
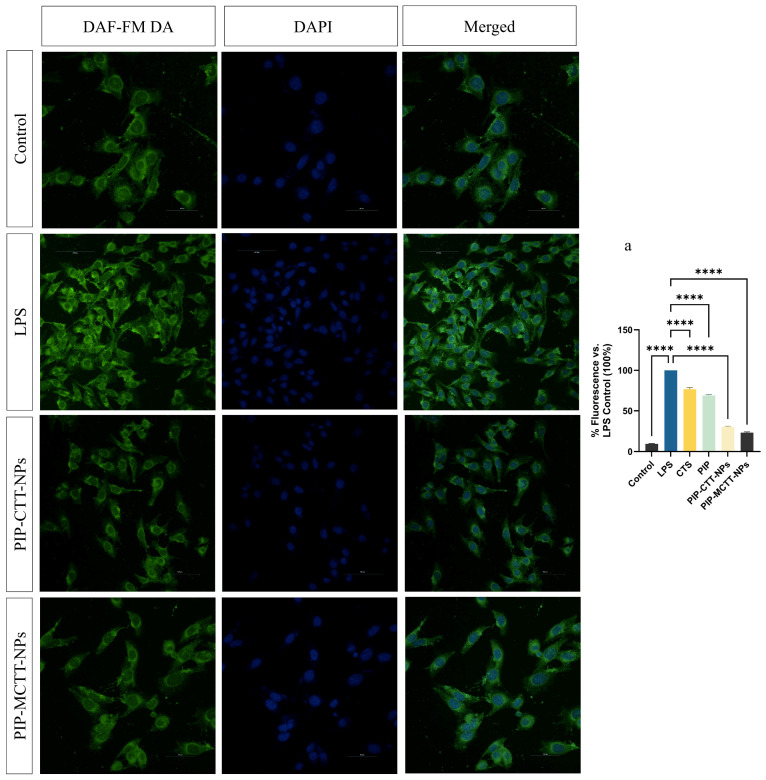
Confocal microscopy images and (**a**) quantitative analysis of intracellular NO inhibition in RAW 264.7 macrophages after LPS stimulation and treatment with CTS, PIP, PIP-CTT-NPs, and PIP-MCTT-NPs. Images for CTS and free PIP treatments are omitted for clarity. Scale bar = 100 µm. Fluorescence intensity is expressed as percentage (%) relative to LPS control (set as 100%). Data are presented as mean ± SD (n = 3 independent biological experiments). **** indicates *p* < 0.0001 compared to LPS control The apparent differences in nuclear size among representative images are due to image cropping and field selection during figure preparation; all images were acquired under identical magnification and imaging conditions.

**Figure 14:**
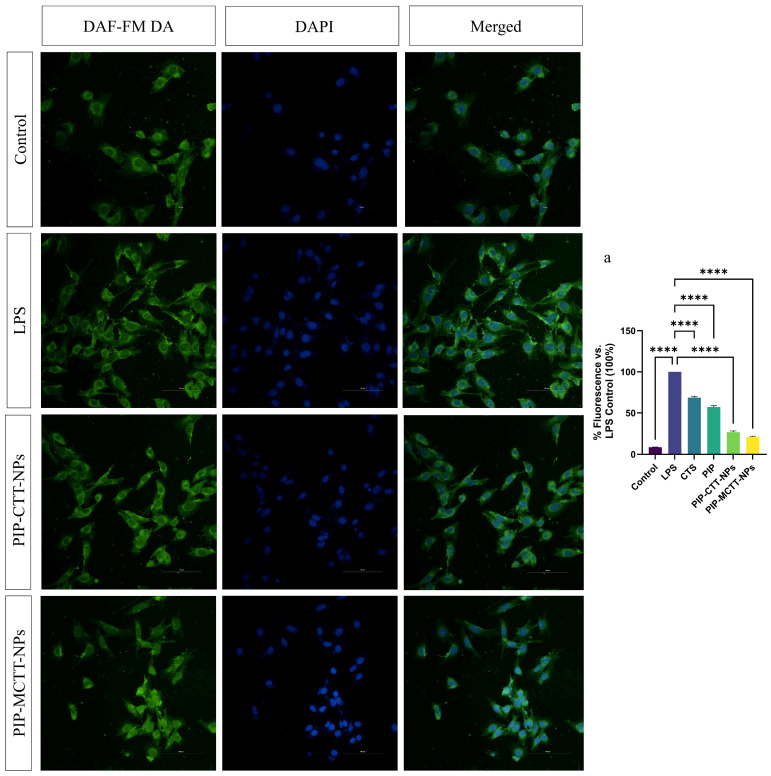
Confocal microscopy images and (**a**) quantitative analysis of intracellular NO inhibition in THP-1 macrophages after LPS stimulation and treatment with CTS, PIP, PIP-CTT-NPs, and PIP-MCTT-NPs. Images for CTS and free PIP treatments are omitted for clarity. Scale bar = 100 µm. Fluorescence intensity is expressed as percentage (%) relative to LPS control (set as 100%). Data are presented as mean ± SD (n = 3 independent biological experiments). **** indicates *p* < 0.0001 compared to LPS control.

Confocal microscopy coupled with CTCF quantification revealed a marked rise in nitric oxide levels following LPS stimulation in both RAW 264.7 and THP-1 macrophages, with signal intensity normalized to 100% (Figure 13 and Figure 14). Minimal green signal (9%) was observed in untreated controls, confirming low basal NO production. Treatment with CTS and free PIP partially reduced the fluorescence signal to 71% and 60% in RAW 264.7 cells, and 67% and 56% in THP-1 cells, respectively (images omitted for clarity). In contrast, nanoparticle treatments produced a much stronger reduction.

The non-targeted PIP-CTT-NPs lowered signal intensity to 38% in RAW 264.7 and 35% in THP-1 macrophages, whereas the mannose-engineered PIP-MCTT-NPs achieved the most significant decrease, reaching 25% and 27% of the LPS control, respectively. Cell number-independent quantification via DAPI-normalized CTCF (Table S2) verified that mannose-targeted PIP-MCTT-NPs achieved 52–58% greater intracellular NO inhibition compared to free piperine. This clearly indicates that nanoencapsulation markedly enhances the intracellular suppression of nitric oxide, likely due to improved uptake and retention within the macrophages [42,47,48]. The mannose-modified formulation demonstrated the most effective inhibition, underscoring the role of receptor-mediated internalization in strengthening anti-inflammatory activity. Such a pronounced intracellular reduction by a piperine-loaded, TPGS/Tween-stabilized delivery system has not been previously described, emphasizing its distinctive potential for macrophage-targeted anti-inflammatory therapy. Duan et al. previously reported that free piperine reduced reactive oxygen species by 63.12% in RAW 264.7 cells [87]. In comparison, our mannose-modified nanocarriers achieved a 73–75% decline in intracellular NO (from 100% to 25–27%), reflecting a substantially greater cellular response. This enhanced effect likely arises from the synergistic influence of mannose-mediated targeting and the solubilizing action of TPGS and Tween 80, resulting in superior intracellular transport compared to unencapsulated piperine.

##### Pro-Inflammatory Cytokine Inhibition

**Figure 15:**
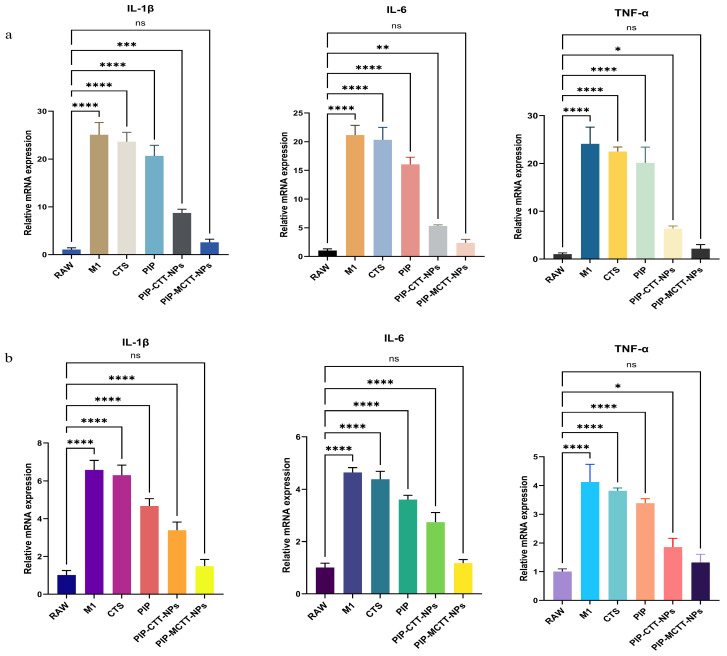
Effects of CTS, PIP, PIP-CTT-NPs, and PIP-MCTT-NPs (64 µg/mL) on macrophage polarization in vitro. qRT-PCR analysis of M1-associated genes IL-1β, IL-6, and TNF-α in (**a**) RAW 264.7 and (**b**) THP-1 macrophages. Data are presented as mean ± SD (n = 3 independent biological experiments). Statistical analysis was performed using one-way ANOVA. *p* < 0.05 was considered statistically significant. ns indicates no significant difference compared with control; * *p* < 0.05, ** *p* < 0.01, *** *p* < 0.001, **** *p* < 0.0001.

Quantitative real-time PCR analysis revealed a pronounced induction of IL-1β, IL-6, and TNF-α transcripts in LPS-stimulated RAW 264.7 and THP-1 macrophages, consistent with the elevated NO production observed in earlier assays (Figure 15). Treatment with CTS and free PIP moderately attenuated this response, yielding only partial reductions in inflammatory signaling (20–36% inhibition). Encapsulation of piperine within nanoparticles markedly enhanced the anti-inflammatory outcome. The non-targeted PIP-CTT-NPs further reduced mRNA levels of all three cytokines, in agreement with their stronger suppression of both extracellular and intracellular NO. The mannose-modified nanocarriers (PIP-MCTT-NPs) achieved the most substantial downregulation, exhibiting the lowest transcript abundance among all treatment groups. This enhanced regulatory effect reflects the benefits of receptor-mediated internalization combined with TPGS/Tween 80–based stabilization, which improves intracellular drug delivery and retention [37,47].

Compared with previous studies, the current formulation displayed superior potency. Duan et al. reported that free piperine (20 mg/L) reduced TNF-α, IL-1β, and IL-6 transcription by 35.89%, 66.04%, and 46.87%, respectively, in LPS-stimulated RAW 264.7 cells [87]. In contrast, our mannose-engineered nanoparticles achieved greater cytokine suppression at lower concentrations, demonstrating the synergistic advantage of targeted nanocarrier delivery. Similarly, Bang et al. observed anti-inflammatory activity of piperine in arthritis models [20]; however, the macrophage-specific uptake conferred by mannose decoration and surfactant stabilization in our system significantly amplifies this therapeutic effect. Collectively, these findings highlight the clinical potential of the PIP-MCTT-NP formulation as a macrophage-directed anti-inflammatory platform that surpasses the efficacy of conventional piperine treatments.

It should be noted that the present anti-inflammatory evaluation is based on functional inhibition of nitric oxide and transcriptional downregulation of pro-inflammatory cytokines. Although these outcomes are consistent with reported NF-κB/MAPK-mediated mechanisms of piperine action [87,96], protein-level validation via Western blotting (NF-κB/MAPK phosphorylation) was prioritized for our ongoing in vivo investigations rather than the current in vitro scope. These analyses (IKK/p-IKK, p65/p-p65, p-JNK/JNK, etc.) will provide comprehensive mechanistic confirmation in future publications.

#### 3.2.7. Antioxidant Evaluation

##### Evaluation of Antioxidant and Reducing Potential

**Figure 16:**
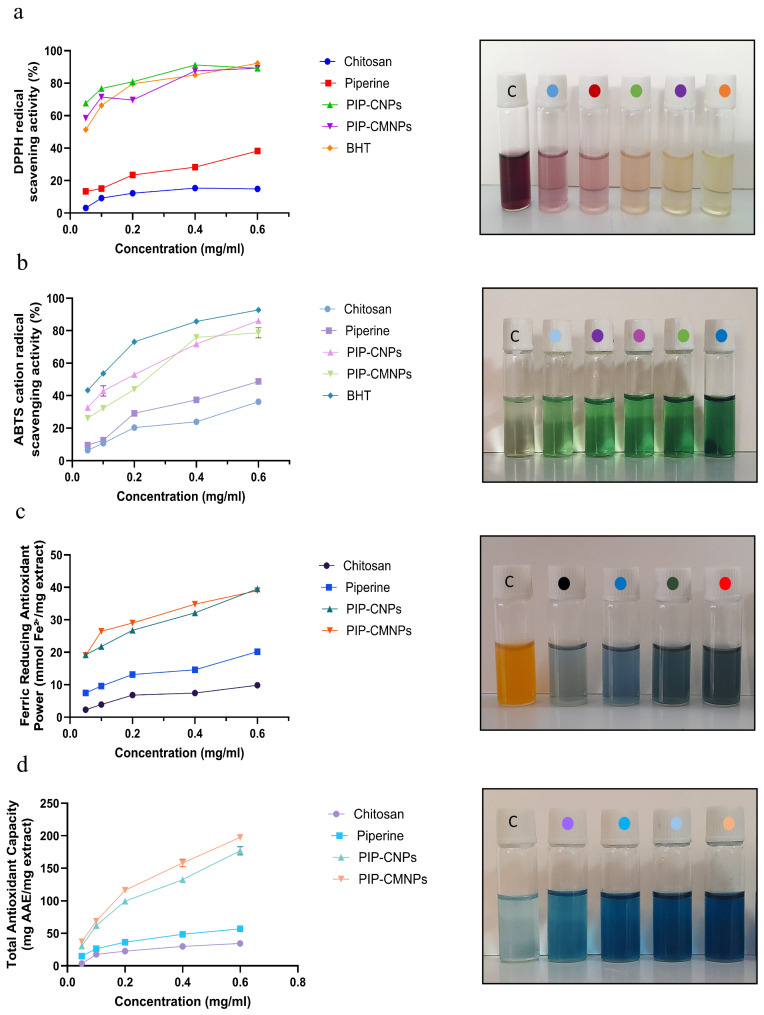
In vitro antioxidant activities of CTS, PIP, PIP-CTT-NPs, and PIP-MCTT-NPs compared with standard antioxidants. BHT was used as the standard for DPPH and ABTS assays, FeSO_4_ for the FRAP assay, and ascorbic acid for the TAC assay. (**a**) DPPH, (**b**) ABTS, (**c**) FRAP, and (**d**) TAC assays. Data are presented as mean ± SEM (n = 3 independent experiments). Statistical analysis was performed using two-way ANOVA (*p* < 0.001).

Various in vitro assays, including DPPH, ABTS•+, FRAP, and TAC, were used to evaluate the antioxidant activity of CTS, PIP, PIP-CTT-NPs, and PIP-MCTT-NPs at different concentrations ranging from 0.05 to 0.6 mg/mL. The antioxidant effects of these samples were compared with standard antioxidants (BHT, FeSO_4_, and ascorbic acid), which served as reference controls [97,98]. The results are presented in Figure 16.

In both the DPPH and ABTS assays, CTS and PIP exhibited lower antioxidant activities than BHT across all tested concentrations, with statistically significant differences (*p* < 0.05). In contrast, nanoparticle formulations markedly enhanced antioxidant effects. In the DPPH assay, PIP-CTT-NPs and PIP-MCTT-NPs displayed comparable or even higher activity than BHT, with effects increasing dose-dependently. Specifically, the antioxidant activity of BHT ranged from 57.14% to 92.53%, while PIP-CTT-NPs showed 67.69–89.24% activity and PIP-MCTT-NPs ranged from 58.42 to 89.16%. At concentrations of 0.05, 0.1, 0.2, and 0.4 mg/mL, PIP-CTT-NPs outperformed BHT in the DPPH assay, while at 0.6 mg/mL the activity was comparable. PIP-MCTT-NPs exceeded BHT at 0.05, 0.1, and 0.4 mg/mL and showed similar activity at 0.2 and 0.6 mg/mL. PIP-CTT-NPs achieved their peak scavenging activity at 0.4 mg/mL, whereas PIP-MCTT-NPs reached their maximum at 0.6 mg/mL (Figure 16a).

In the ABTS assay, although the activities of PIP-CTT-NPs and PIP-MCTT-NPs were slightly lower than that of BHT, both formulations showed a clear dose-dependent increase. At 0.6 mg/mL, PIP-CTT-NPs and PIP-MCTT-NPs achieved their highest ABTS•+ scavenging activities, measuring 86.16 ± 0.357% and 78.68 ± 3.085%, respectively. Overall, nanoparticle formulations approached the activity of BHT, while CTS and PIP remained the least effective (Figure 16b). In the FRAP assay, PIP-CTT-NPs and PIP-MCTT-NPs exhibited significantly greater ferric reducing power than CTS and PIP across all concentrations. Similarly, in the TAC assay, both nanoparticle systems consistently showed higher total antioxidant capacity compared to CTS and PIP. These findings highlight that encapsulation of PIP within TPGS/Tween 80-stabilized chitosan nanoparticles significantly improved its antioxidant efficiency, consistent with the trends observed in the DPPH and ABTS assays (Figure 16c,d). All four antioxidant assays exhibited distinct color transitions with increasing sample concentration, where deeper coloration corresponded to stronger radical-scavenging effects, validating the quantitative trends (Figure 16a–d) [99].

Notably, despite the sustained release profile observed in Figure 8, the enhanced antioxidant activity can be attributed to the improved surface accessibility and dispersion of piperine within the nanoparticle system. A fraction of piperine remains readily available at or near the nanoparticle interface, enabling rapid interaction with reactive species without requiring complete release. The enhanced performance of PIP-CTT-NPs and PIP-MCTT-NPs arises from the combined influence of TPGS and Tween 80, which improve solubility and stability of piperine, thereby amplifying its redox potential [39,43,47,83]. Mannose decoration further promoted nanoparticle dispersion and effective interaction with reactive species, augmenting free radical neutralization compared with CTS and unencapsulated PIP [100]. The collective outcomes of the DPPH, ABTS, FRAP, and TAC assays demonstrate that the mannose-engineered, TPGS/Tween-stabilized nanocarriers provide markedly superior antioxidant capacity relative to free or non-encapsulated counterparts. This study highlights that surfactant stabilization and receptor-targeted functionalization can synergistically enhance piperine oxidative defense properties, underscoring the therapeutic potential of this nanosystem in antioxidant nanomedicine.

Our findings also surpass previously reported piperine data. Kusumorini et al. observed limited DPPH activity for isolated piperine, with ABTS IC_50_ values of 4.35 ± 0.004 mg/mL and FRAP values of 10.53 ± 0.06 mol TE/g sample [101]. In comparison, our PIP-CTT-NPs and PIP-MCTT-NPs displayed DPPH scavenging efficiencies of 67.69–89.24% and 58.42–89.16%, respectively, often outperforming the standard antioxidant BHT. Likewise, Al-Khayri et al. reported only 19.5% DPPH inhibition for *Piper nigrum* extracts, whereas our nanoformulations exhibited substantially greater radical-scavenging potency [102]. These improvements highlight the pivotal contribution of TPGS/Tween 80 stabilization in overcoming piperine’s inherent solubility and bioavailability limitations.

##### Evaluation of Intracellular ROS and RNS Modulation

**Figure 17:**
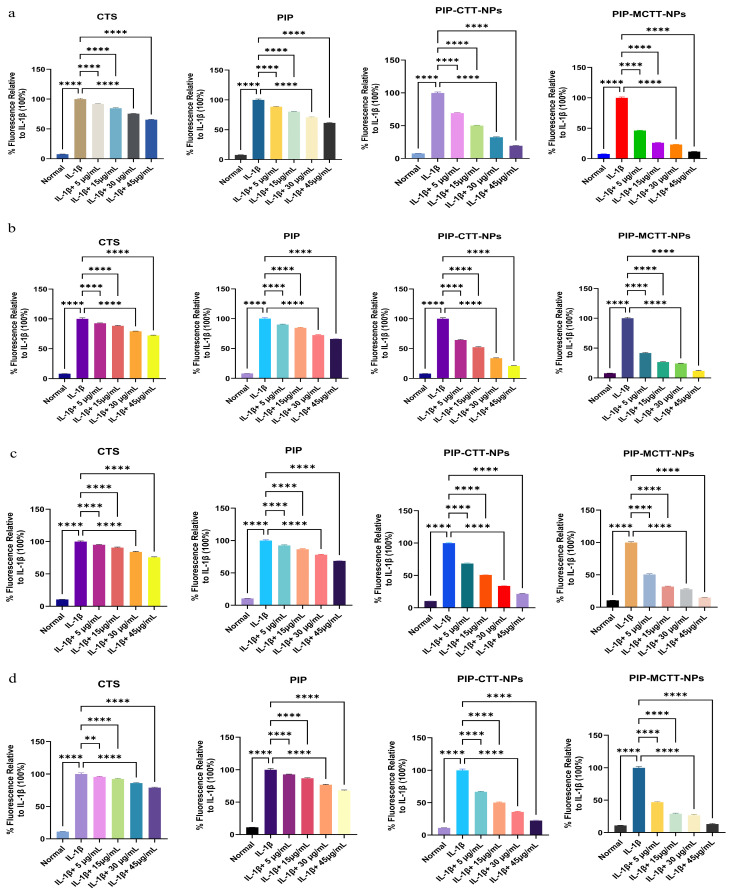
Intracellular ROS and RNS modulation by CTS, PIP, and nanoparticle formulations at different concentrations. (**a**) ROS levels in RAW 264.7 cells, (**b**) RNS levels in RAW 264.7 cells, (**c**) ROS levels in THP-1 cells, and (**d**) RNS levels in THP-1 cells. Cells were stimulated with IL-1β prior to treatment. Data are presented as mean ± SD (n = 3 independent biological experiments). Statistical analysis was performed using one-way ANOVA. ** *p* < 0.01; **** *p* < 0.0001 compared with the IL-1β-stimulated control.

The intracellular ROS and RNS levels in RAW 264.7 and THP-1 cells after treatment with CTS, PIP, non-targeted nanoparticles, and mannose-targeted nanoparticles are summarized in Figure 17. As expected, IL-1β stimulation significantly elevated both ROS and RNS compared with the untreated control, confirming successful induction of oxidative and nitrosative stress in both cell lines [103,104].

In RAW 264.7 cells, CTS produced a modest reduction in ROS (34%) and RNS (27.6%) at the highest dose (45 µg/mL), indicating limited scavenging capacity. Free PIP displayed a moderate effect, reducing ROS by 39% and RNS by 34%, consistent with its inherent radical-neutralizing properties. A more notable improvement was observed with PIP-CTT-NPs, which suppressed ROS by 81% and RNS by 79%. This marked enhancement can be attributed to the stabilizing and solubilizing role of TPGS and Tween 80 within the chitosan nanoparticles, which improve the intracellular delivery and retention of PIP [105,106]. The mannose-targeted PIP-MCTT-NPs achieved the most pronounced effects, with 88% inhibition of both ROS and RNS, reflecting superior uptake via mannose receptor-mediated endocytosis in addition to the formulation benefits of TPGS and Tween 80 (Figure 17a,b) [37,107].

Similar trends were evident in THP-1 cells, although the overall levels of inhibition were slightly lower than in RAW 264.7. CTS again showed weak activity, reducing ROS (24%) and RNS (21%), while free PIP exerted a moderate inhibition (31% ROS, 32% RNS). PIP-CTT-NPs markedly enhanced activity, suppressing 78% of both ROS and RNS, whereas PIP-MCTT-NPs reached 85–87% inhibition, confirming their improved targeting efficiency (Figure 17c,d).

Overall, the results revealed a clear hierarchy of antioxidant efficacy: PIP-MCTT-NPs > PIP-CTT-NPs > free piperine > chitosan (CTS). The TPGS/Tween 80-stabilized nanoparticles improved the solubility, stability, and sustained release of piperine, while mannose functionalization provided an additional advantage by enhancing macrophage-specific uptake. The dual suppression of reactive oxygen species (ROS) and reactive nitrogen species (RNS)—with inhibition levels of 88% and 86%, respectively—effectively mitigated oxidative and nitrosative stress. This combination protected macrophages from inflammatory damage. These findings aligned with earlier studies showing that ligand-targeted and surfactant-stabilized nanocarriers enhance antioxidant and anti-inflammatory performance compared to free phytochemicals [37,70]. Importantly, this study provided the first evidence of dual ROS/RNS suppression exceeding 85% in macrophages using mannose-functionalized, TPGS/Tween 80-stabilized piperine nanoparticles, underscoring their novelty and therapeutic promise.

The observed antioxidant potency represented a clear advancement over previous reports. Sheweita et al. [108] reported that Tween 80-coated chitosan nanoparticles increased antioxidant enzyme activities (GST, GPx, GR, CAT, and SOD), while Gopalakrishnan et al. [109] demonstrated improved efficacy of ellagic acid using similar systems. Likewise, Yu et al. [110] found that mannosylated nanoparticles enhanced cellular delivery by fourfold in macrophages. In comparison, our PIP-MCTT-NPs achieved superior intracellular antioxidant activity (88% ROS and 86% RNS inhibition) relative to non-targeted systems (81% and 79%), confirming the synergistic benefits of mannose targeting with TPGS/Tween 80 stabilization for enhanced intracellular antioxidant delivery.

## 4. Conclusions

Piperine-loaded mannosylated chitosan nanoparticles were successfully synthesized by ionic gelation and emulsification, with TPGS and Tween 80 as stabilizers. Physico-chemical and spectroscopic analyses confirmed stable nanoparticle formation, with targeted formulations showing larger particle size and rougher morphology. Both targeted and non-targeted systems demonstrated high encapsulation efficiency, stability, and sustained PIP release over 72 h. Importantly, mannose-functionalized nanoparticles significantly enhanced cellular uptake in macrophages compared to non-targeted ones.

Biological evaluations revealed that both formulations reduced extracellular and intracellular nitric oxide levels and downregulated pro-inflammatory cytokines, with stronger suppression observed in targeted nanoparticles. Antioxidant assays and ROS/RNS measurements further confirmed their ability to mitigate oxidative stress in a dose-dependent manner. Apoptosis analysis using Annexin V–FITC/PI staining demonstrated dose-dependent induction of apoptosis at higher concentrations, while maintaining acceptable biocompatibility at concentrations ≤ 64 μg/mL. The enhanced apoptotic response observed in targeted nanoparticles further supports improved intracellular delivery efficiency. Collectively, these findings demonstrate that mannosylated chitosan nanoparticles can overcome PIP solubility and bioavailability limitations while enhancing its anti-inflammatory and antioxidant potential. This formulation strategy highlights a promising approach for targeted delivery of piperine to macrophages, with potential therapeutic implications for inflammation-associated disorders. Future in vivo studies are warranted to validate the translational potential and evaluate long-term safety and efficacy.

## Figures and Tables

**Table 1:** Composition and Preparation Parameters of Nanoparticle Formulations.

Formulation Code	Chitosan (mg)	TPGS (mg)	Tween 80 (µL)	Mannose (mg)	Piperine (mg)	CM6 (mg)	STPP (mg)
PIP-CTT-NPs	20	15	100	–	2.5	–	2
PIP-MCTT-NPs	20	15	100	8	2.5	–	2
CM6-CTT-NPs	20	15	100	–	–	0.2	2
CM6-MCTT-NPs	20	15	100	8	–	0.2	2

“–” indicates the absence of the component in the formulation.

**Table 2:** Experimental Conditions for Antioxidant Assays.

Assay	Reagents & Conditions	Incubation/Time	Wavelength (nm)	Standard	Calculation
DPPH	0.1 mM DPPH	30 min, dark	517 nm	BHT	% Radical Scavenging Equation (7); [66].
ABTS	7 mM ABTS + 2.45 mM K_2_S_2_O_8_	15 min, dark	734 nm	BHT	% Radical Scavenging Equation (7); [67].
FRAP	FRAP solution (prepared from FeSO_4_)	10 min at 37 °C	593 nm	FeSO_4_ standard curve	mmol Fe(II)/mg extract [68].
TAC	Phosphomolybdate reagent	90 min at 95 °C	695 nm	Ascorbic acid	ppm ascorbic acid equivalents [69].

**Table 3:** CHNS Analysis and Substitution Degree.

Samples	Elemental Analysis					DS (%)
	Carbon (%)	Hydrogen (%)	Nitrogen (%)	Sulfur (%)	Oxygen (%)	
CTS	48.18	8.12	7.65	0.03	38.72	-
MNS	42.35	12.55	0.02	0.00	39.19	-
PIP	71.56	6.71	4.91	0.00	16.82	-
PIP-CTT-NPs	49.66	7.61	6.51	0.02	35.72	-
PIP-MCTT-NPs	45.14	9.11	3.22	0.04	44.08	50.73

**Table 4:** Physicochemical Characteristics of the Formulated Nanoparticles.

Formulation Code	Particle Size (nm)	Polydispersity	Zetapotential (mV)	Entrapment Efficiency (%)
PIP-CTT-NPs	78.02 ± 1.20	0.265 ± 0.004	10.61 ± 0.586	92.48 ± 1.56
PIP-MCTT-NPs	162.65 ± 0.66	0.288 ± 0.003	9.34 ± 0.098	82.32 ± 2.790
CM6-CTT-NPs	76.07 ± 0.29	0.196 ± 0.002	2.11 ± 0.007	92.18 ± 2.727
CM6-MCTT-NPs	160.18 ± 1.93	0.216 ± 0.007	1.78 ± 0.332	80.25 ± 1.623

Results are presented as mean ± standard deviation, calculated from triplicate experiments (n = 3).

**Table 5:** DLS measurements of nanoparticle size and stability at different time points.

Batch	Particle Diameter (nm)			PDI (Polydispersity Index)			Surface Charge (mV)		
				Time (days)					
	0	10	30	0	10	30	0	10	30
1	78.02 ± 1.2	79.5 ± 0.2	80.06 ± 0.7	0.265 ± 0.4	0.262 ± 0.7	0.263 ± 0.2	10.6 ± 0.5	10.8 ± 0.1	10.9 ± 0.17
2	162.6 ± 0.6	163.7 ± 0.4	164.8 ± 0.6	0.288 ± 0.3	0.290 ± 0.4	0.291 ± 0.7	9.3 ± 0.9	9.2 ± 0.2	9.2 ± 0.19

Results for (1) PIP-CTT-NPs and (2) PIP-MCTT-NPs are expressed as mean ± SD (n = 3).

## Data Availability

The data presented in this study are available in the article and Supplementary Materials.

## Supplementary Materials

The following supporting information can be downloaded at: https://www.mdpi.com/article/10.3390/antiox15050559/s1, Figure S1: Graph illustrating the preparation methods for (A) Piperine/Coumarin-6-loaded chitosan-TPGS-Tween 80 nanoparticles (PIP-CTT-NPs) and (B) Mannose-conjugated Piperine/Coumarin-6-loaded chitosan-TPGS-Tween 80 nanoparticles (PIP-MCTT-NPs); Table S1: List of Primers Used for Pro-inflammatory Cytokine Genes in Macrophages; Table S2: Normalized CTCF Quantification Confirming Cell Number-Independent Superiority of Mannose-Targeted Nanoparticles; Figure S2: Cell proliferation activity assay in THP-1 cells following PMA treatment. (A) The effects of PMA on THP-1 cell viability were assessed using the MTT assay. THP-1 cells were treated with varying concentrations of PMA (0, 25, 50, 100, 200, 300, and 400 ng/mL), and cell viability was evaluated after exposure. The MTT assay results showed a dose-dependent effect of PMA on THP-1 cell viability, with increased cell viability observed at lower concentrations and decreased viability at higher concentrations, as visualized under a light microscope. (B) Cell proliferation results are shown for THP-1 cells treated with PMA. **** represents *p* < 0.0001; Figure S3: Morphological comparison between THP-1 monocytes and their macrophage-like derivatives during PMA-induced differentiation at the resting stage (M0). THP-1 cells were seeded at a density of 1 × 10^5^ cells per well in a 6-well clear-bottom plate and treated with 200 ng/mL PMA or a DMSO control. Cells were imaged at 0, 6, 12, and 24 h during treatment. Yellow arrows indicate differentiated cells exhibiting an adherent and flattened morphology. Imaging was performed using a light microscope with 4×, 10×, and 20× objectives for detailed analysis; Figure S4. Effect of different concentrations of CTS, PIP, PIP-CTT-NPs, and PIP-MCTT-NPs on the viability of (a) RAW 264.7 and (b) THP-1 macrophage cells evaluated by the MTT assay. Data are expressed as percentage (%) cell viability relative to the untreated control (set at 100%) and presented as mean ± SD (n = 3 independent biological experiments, each performed in technical triplicates). Statistical significance was analyzed using one-way ANOVA. *p* < 0.05 was considered statistically significant. ns indicates no significant difference compared with control; *p* < 0.05, * *p* < 0.01, ** *p* < 0.001, *** *p* < 0.0001; Figure S5. Annexin V–FITC/PI apoptosis analysis of THP-1 macrophages treated with free PIP and nanoparticle formulations. Representative flow cytometry dot plots are shown for (a) untreated control cells; (b–d) cells treated with free PIP, PIP-CTT-NPs, and PIP-MCTT-NPs, respectively, at 64 μg/mL; and (e–g) cells treated with free PIP, PIP-CTT-NPs, and PIP-MCTT-NPs, respectively, at 128 μg/mL. Quantitative analysis of total apoptotic cells (early + late apoptosis) is presented in (h) for 64 μg/mL and (i) for 128 μg/mL. Data are expressed as mean ± SD (n = 3 independent experiments).
