# Supplementary material for: Mannose-Functionalized Chitosan-TPGS/Tween 80 Nanocarriers for Macrophage Targeting: Enhanced Piperine Delivery to Potentiate Anti-Inflammatory and Antioxidant Therapy
Source: Antioxidants (Basel). 2026 Apr 28;15(5):559. doi: 10.3390/antiox15050559 (PMC13203094; doi:10.3390/antiox15050559)
Supplement: Supplementary file 1 [file antioxidants-15-00559-s001.zip › antioxidants-4214827-supplementary.pdf]

Supplemental material

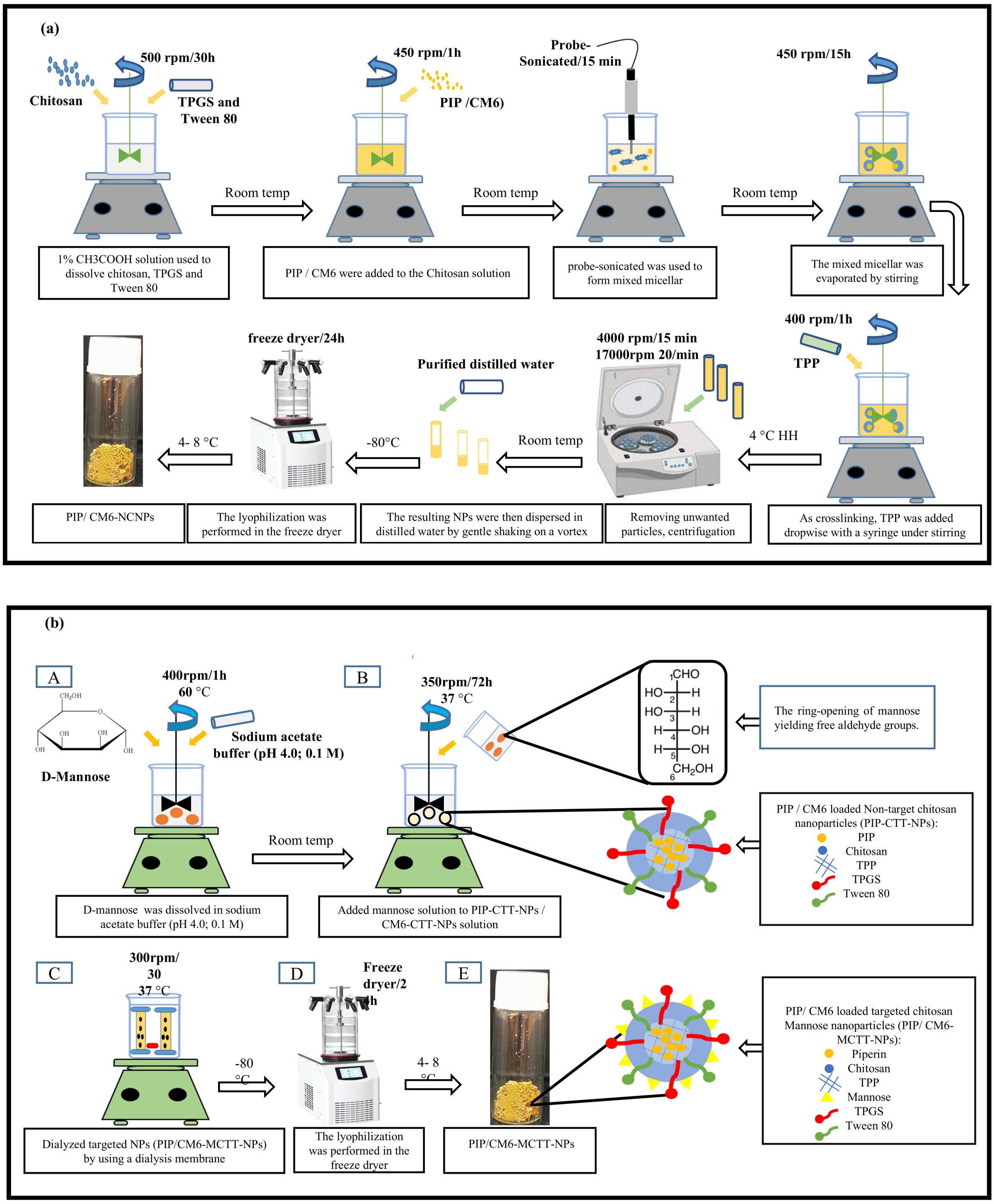

Figure. S1 Graph illustrating the preparation methods for (A) Piperine/Coumarin-6-loaded chitosan-TPGS-Tween 80 nanoparticles (PIP-CTT-NPs) and (B) Mannose-conjugated Piperine/Coumarin-6-loaded chitosan-TPGS-Tween 80 nanoparticles (PIP-MCTT-NPs)

Table S1 List of Primers Used for Pro-inflammatory Cytokine Genes in Macrophages

Gene	Primer
**β-actin-F**	5′-CGGTGCAGACATTTGGTGGG-3′
**β-actin-R**	5′-AGG CCATAAGGGAAGGGACA-3′
**Il-6-F**	5′-CAACGATGATGCACTTGCAGA-3′
**Il-6-R**	5′-TGTGACTCCAGCTTATCTCTTGG-3′
**Il-1β-F**	5′- TTCAAGGGGACATTAGGCAG-3′
**Il-1β-R**	5′-TGTGCTGGTGCTTCATTCAT-3′
**Tnf-α-F**	5′- CTCAGCGAGGACAGCAAGG-3′
**Tnf-α-R**	5′-AGGGACAGAACCTGCCTGG-3′

Table S2. Normalized CTCF Quantification Confirming Cell Number-Independent Superiority of Mannose-Targeted Nanoparticles:

Figure	Cell Line	Parameter	Free CM6/PIP	CTT-NPs	MCTT-NPs	MCTT vs CTT	MCTT vs Free
Fig 10	RAW264.7	CTCF/nucleus (%)	100%	351%	591%	1.7x↑	5.9↑
Fig 11	THP-1	CTCF/nucleus (%)	100%	368%	577%	1.6x↑	5.8↑
Fig 13	RAW264.7	NO-CTCF/nucleus (%)	60%	38%	25%	↓34%	↓58%
Fig 14	THP-1	NO-CTCF/nucleus (%)	56%	35%	27%	↓23%	↓52%

CTCF values normalized to DAPI-stained nuclei count from n=3 independent experiments (1×10^5 cells/well seeding density). MCTT-NPs demonstrate 5.8-5.9× greater uptake and 52-58% superior NO inhibition versus free drug, confirming quantitative results independent of field-to-field cell number variation ($p<0.01$).

A

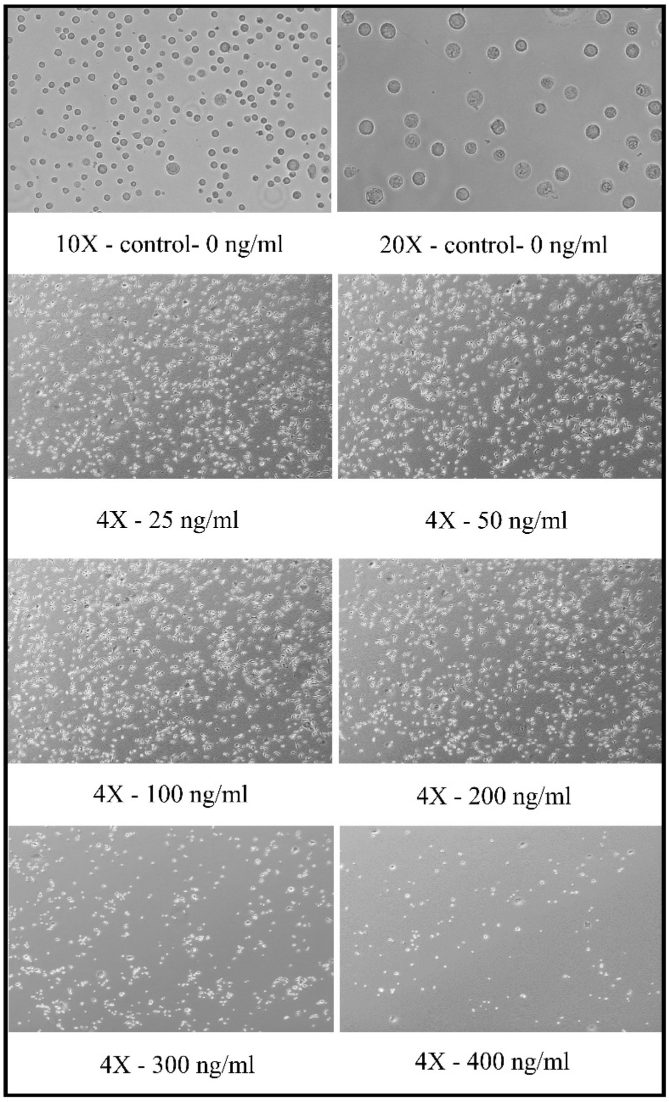

B

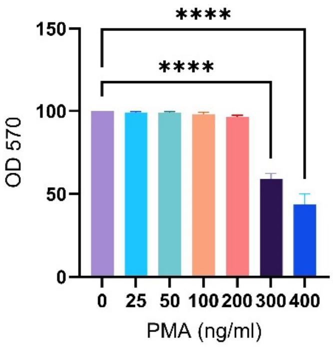

Figure S2 Cell proliferation activity assay in THP-1 cells following PMA treatment. (A) The effects of PMA on THP-1 cell viability were assessed using the MTT assay. THP-1 cells were treated with varying concentrations of PMA (0, 25, 50, 100, 200, 300, and 400 ng/mL), and cell viability was evaluated after exposure. The MTT assay results showed a dose-dependent effect of PMA on THP-1 cell viability, with increased cell viability observed at lower concentrations and decreased viability at higher concentrations, as visualized under a light microscope. (B) Cell proliferation results are shown for THP-1 cells treated with PMA.**** represents $p < 0.0001$.

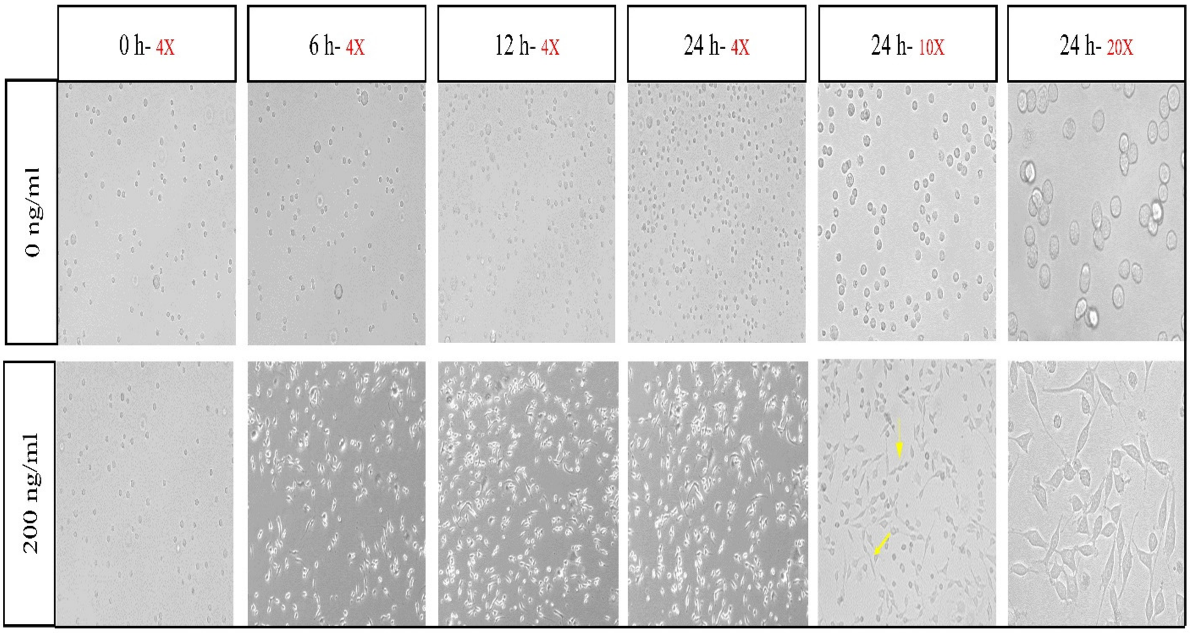

Figure S3 Morphological comparison between THP-1 monocytes and their macrophage-like derivatives during PMA-induced differentiation at the resting stage (M0). THP-1 cells were seeded at a density of 1 × 10^5 cells per well in a 6-well clear-bottom plate and treated with 200 ng/mL PMA or a DMSO control. Cells were imaged at 0-, 6-, 12-, and 24-hours during treatment. Yellow arrows indicate differentiated cells exhibiting an adherent and flattened morphology. Imaging was performed using a light microscope with 4×, 10×, and 20× objectives for detailed analysis.

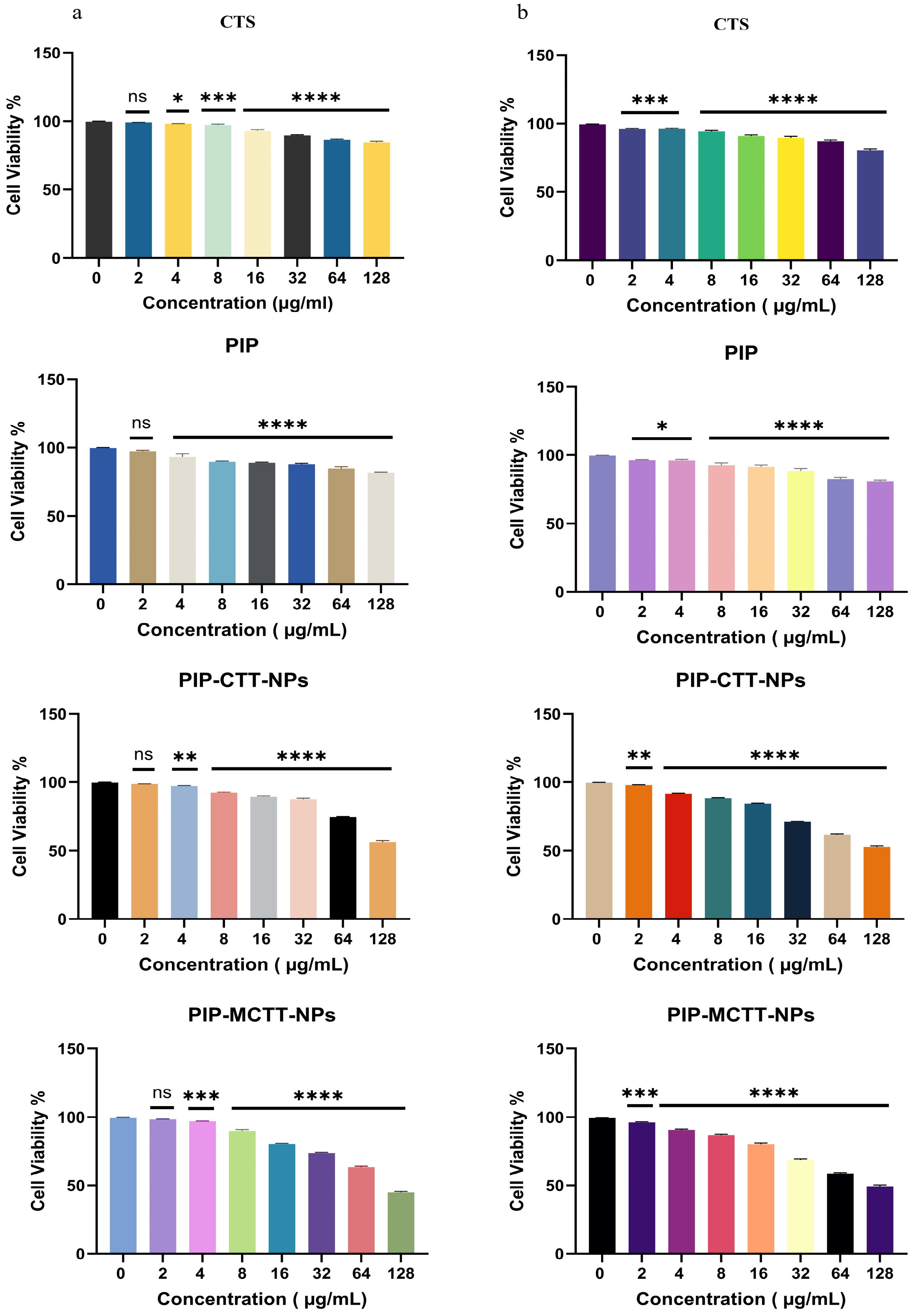

a
CTS
Cell Viability %
150
100
50
0
ns
*
***
****
0
2
4
8
16
32
64
128
Concentration (μg/ml)
PIP
ns
****
Concentration (μg/mL)
PIP-CTT-NPs
ns
**
****
Concentration (μg/mL)
PIP-MCTT-NPs
ns
***
****
Concentration (μg/mL)
b
CTS
***
****
Concentration (μg/mL)
PIP
*
****
Concentration (μg/mL)
PIP-CTT-NPs
**
****
Concentration (μg/mL)
PIP-MCTT-NPs
***
****
Concentration (μg/mL)

Figure S4. Effect of different concentrations of CTS, PIP, PIP-CTT-NPs, and PIP-MCTT-NPs on the viability of (a) RAW 264.7 and (b) THP-1 macrophage cells evaluated by the MTT assay. Data are expressed as percentage (%) cell viability relative to the untreated control (set at 100%) and presented as mean ± SD (n = 3 independent biological experiments, each performed in technical triplicates). Statistical significance was analyzed using one-way ANOVA. $p < 0.05$ was considered statistically significant. ns indicates no significant difference compared with control; $p < 0.05$, *$p < 0.01$, **$p < 0.001$, ***$p < 0.0001$.

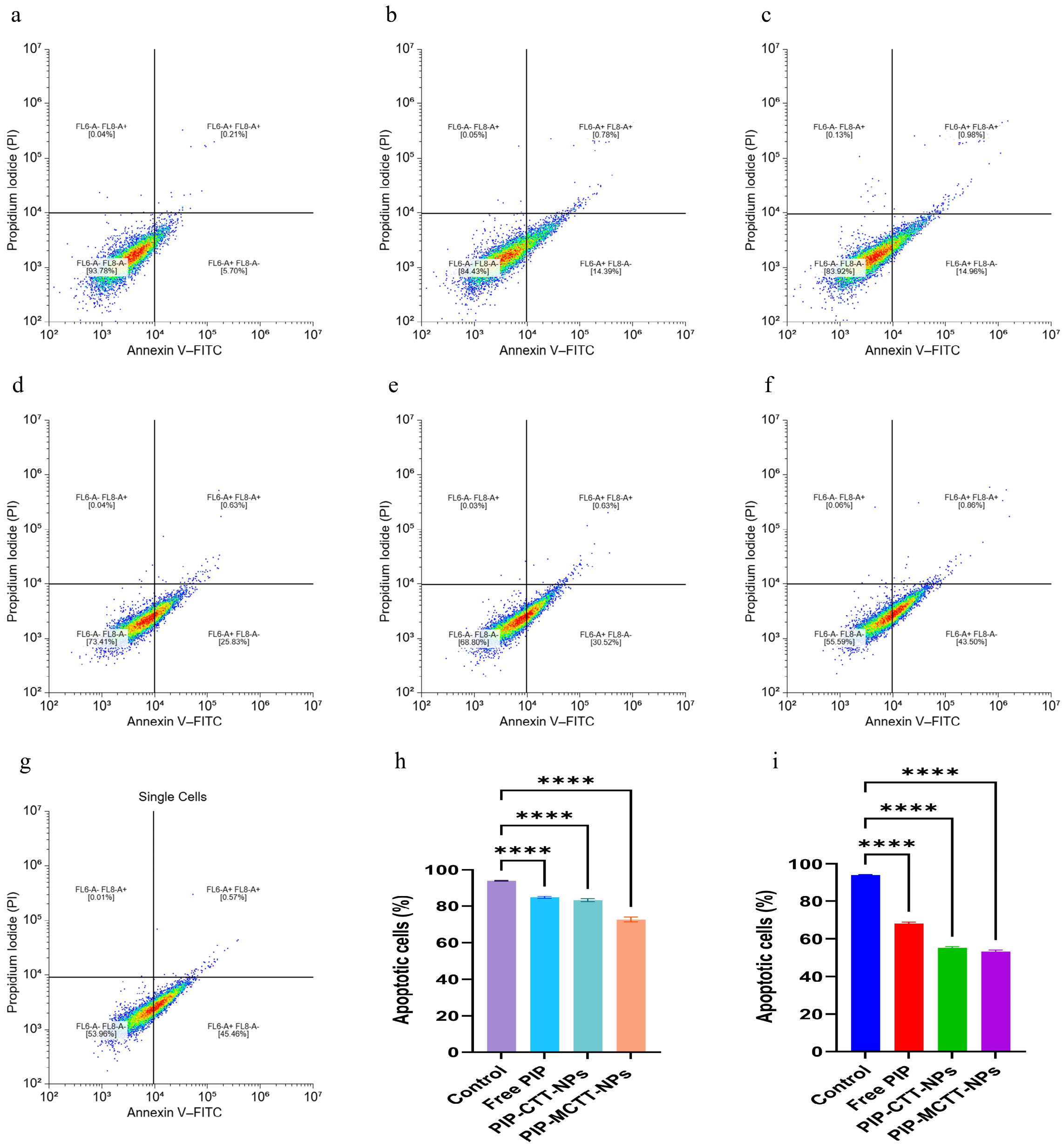

Figure S5. Annexin V–FITC/PI apoptosis analysis of THP-1 macrophages treated with free PIP and nanoparticle formulations. Representative flow cytometry dot plots are shown for (a) untreated control cells; (b–d) cells treated with free PIP, PIP-CTT-NPs, and PIP-MCTT-NPs, respectively, at 64 μg/mL; and (e–g) cells treated with free PIP, PIP-CTT-NPs, and PIP-MCTT-NPs, respectively, at 128 μg/mL. Quantitative analysis of total apoptotic cells (early + late apoptosis) is presented in (h) for 64 μg/mL and (i) for 128 μg/mL. Data are expressed as mean ± SD (n = 3 independent experiments).
